# BMP Inhibition in Seminomas Initiates Acquisition of Pluripotency via NODAL Signaling Resulting in Reprogramming to an Embryonal Carcinoma

**DOI:** 10.1371/journal.pgen.1005415

**Published:** 2015-07-30

**Authors:** Daniel Nettersheim, Sina Jostes, Rakesh Sharma, Simon Schneider, Andrea Hofmann, Humberto J. Ferreira, Per Hoffmann, Glen Kristiansen, Manel B. Esteller, Hubert Schorle

**Affiliations:** 1 Institute of Pathology, Department of Developmental Pathology, University Medical School, Bonn, Germany; 2 Institute of Human Genetics, University Medical School, Bonn, Germany; 3 Cancer Epigenetics and Biology Program (PEBC), Bellvitge Biomedical Research Institute, L’Hospitalet, Barcelona, Catalonia, Spain; 4 Institute of Pathology, University Medical School, Bonn, Germany; 5 Department of Physiological Sciences II, School of Medicine, University of Barcelona, Barcelona, Catalonia, Spain; 6 Institucio Catalana de Recerca i Estudis Avançats (ICREA), Barcelona, Catalonia, Spain; Brigham and Women's Hospital, UNITED STATES

## Abstract

Type II germ cell cancers (GCC) can be subdivided into seminomas and non-seminomas. Seminomas are similar to carcinoma in situ (CIS) cells, the common precursor of type II GCCs, with regard to epigenetics and expression, while embryonal carcinomas (EC) are totipotent and differentiate into teratomas, yolk-sac tumors and choriocarcinomas. GCCs can present as seminomas with a non-seminoma component, raising the question if a CIS gives rise to seminomas and ECs at the same time or whether seminomas can be reprogrammed to ECs. In this study, we utilized the seminoma cell line TCam-2 that acquires an EC-like status after xenografting into the murine flank as a model for a seminoma to EC transition and screened for factors initiating and driving this process. Analysis of expression and DNA methylation dynamics during transition of TCam-2 revealed that many pluripotency- and reprogramming-associated genes were upregulated while seminoma-markers were downregulated. Changes in expression level of 53 genes inversely correlated to changes in DNA methylation. Interestingly, after xenotransplantation 6 genes (*GDF3*, *NODAL*, *DNMT3B*, *DPPA3*, *GAL*, *AK3L1*) were rapidly induced, followed by demethylation of their genomic loci, suggesting that these 6 genes are poised for expression driving the reprogramming. We demonstrate that inhibition of BMP signaling is the initial event in reprogramming, resulting in activation of the pluripotency-associated genes and NODAL signaling. We propose that reprogramming of seminomas to ECs is a multi-step process. Initially, the microenvironment causes inhibition of BMP signaling, leading to induction of NODAL signaling. During a maturation phase, a fast acting NODAL loop stimulates its own activity and temporarily inhibits BMP signaling. During the stabilization phase, a slow acting NODAL loop, involving WNTs re-establishes BMP signaling and the pluripotency circuitry. In parallel, DNMT3B-driven de novo methylation silences seminoma-associated genes and epigenetically fixes the EC state.

## Author Summary

The understanding of germ cell cancer pathogenesis is based on a linear model, where seminomas and non-seminomas represent distinct entities, although originating from a common precursor lesion, the carcinoma in situ. We demonstrate that germ cell cancer development is a microenvironment-dependent plastic process that allows latent pluripotent seminomas /TCam-2 to acquire primed pluripotency and transit into an EC. For the first time, we show that this plasticity is initiated after interference with BMP signaling and driven by NODAL signaling, which is accompanied by considerable remodeling of the methylome. In conclusion, our data strongly suggest that ECs might also be able to transit into a seminoma upon interference with the drivers of reprogramming identified in this study.

## Introduction

### Typical features of seminomas and embryonal carcinomas

Type II germ cell cancers (GCC) arise from a precursor lesion termed carcinoma in situ (CIS) [1]. CIS cells are thought to be the result of a defective germ cell development and progress into seminomatous and non-seminomatous GCCs [1] [2]. Seminomas grow as a uniform tumor mass and are similar to CIS and PGCs with respect to gene expression. They express PGC- and pluripotency markers like PRDM1 (BLIMP1), TFAP2C, cKIT, SOX17, NANOG and OCT3/4 [1] [3] [4] [5]. Like CIS and PGCs, seminomas display DNA hypomethlyation compared to other GCC entities [6] [7]. Embryonal carcinomas (EC) are totipotent and differentiate into teratomas (cells of all three germ layers), yolk-sac tumors and choriocarcinomas (extra-embryonic tissues) [1]. Further, the DNA of ECs is highly methylated compared to CIS and seminomas [6]. GCCs are termed seminomas, when they consist to 100% of seminoma cells (60.6% of all GCC cases), but GCCs can also present as mixed non-seminomas with or without a seminomatous component (38.8% of all GCC cases), raising the question, if a CIS gives rise to seminomas and ECs at the same time or whether seminomas can be reprogrammed to ECs or vice versa [8] [9] [10]. Generally, seminomas are highly sensitive towards irradiation as well as cisplatin-based chemotherapy [11], while non-seminomas require a more aggressive treatment strategy and are resistant to DNA damage therapies [11] [12]. Thus, a reprogramming of a seminoma to an EC increases the risk of a poor outcome and would make it necessary to adjust the treatment strategy during a patient’s therapy.

### Pluripotency regulation in type II GCCs

Both, seminomas and EC express the pluripotency markers OCT3/4 and NANOG, but expression of the pluripotency factor SOX2 is restricted to ECs, while seminomas express SOX17 instead. Recently, SOX17 has been shown to be a key specifier of the human PGC cell fate by acting upstream of PRDM1 [3] [5] [13] [14]. PRDM1, the transcription factor TFAP2C and SOX17 render the PGCs and seminomas in a state of dormant pluripotency, meaning that they express pluripotency markers, but are not able to induce differentiation into somatic tissues. In contrast, ECs display naïve or primed pluripotency, enabling the cells to differentiate in response to appropriate signals into cells of all germ layers. Furthermore, ECs express several other pluripotency and epigenetic reprogramming factors like REX1 (ZFP42), DPPA3 (STELLA), GDF3, SALL4, PRDM14, DNMT3B /L or ZIC3 [15] [16] [5]. RNAi-mediated knock down of the pluripotency factor *ZIC3* in murine and human ESCs induced *SOX17*, demonstrating that *SOX17* is normally repressed by *ZIC3* [17]. Further, it is known that SOX17 antagonizes WNT signaling, which has been suggested to demarcate seminomas from ECs [18] [19] [20].

### Signaling pathways in normal and malignant germ cells

The members of the TGF-beta superfamily play an important role in regulation of proliferation, differentiation and cell death in a broad variety of cell types and processes, including during PGC formation and GCC pathogenesis [21]. The TGF-beta signaling pathway is activated by binding of its ligands (TGF-betas, Activin /Nodal, GDFs, AMH or BMPs) to a type II receptor (ACVR2A /B, BMPR2, TGF-betaR2) that phosphorylates and activates a type I receptor (ALK3–7: ACVR1B /C, BMPR1A /B, TGF-betaR1) [22]. In turn, type I receptors activate effector moleculs of the SMAD family, which can be subdivided into receptor-SMADS (SMAD1 /2 /3 /5 /9) and co-SMAD (SMAD4) [23]. A complex of R-SMADs and co-SMAD4 acts in the nucleus as transcription factors and regulates target gene expression. A third SMAD class, termed inhibitory-SMADs (SMAD6 /7) is able to counteract these processes. In general, TGF-beta and Activin /Nodal signal via R-SMAD2 /3, while BMPs utilize the R-SMADs 1 /5 /8.

Active NODAL signaling depends on the co-receptor CRIPTO /CRYPTIC and stimulates expression of NODAL as well as LEFTY1 /2, leading to establishment of a signaling loop that stimulates and limits (LEFTY1 /2) itself simultaneously, to prevent an overshooting of mitogenic NODAL signals during embryogenesis or cell differentiation [24] [25] [26] [27]. Active endogenous NODAL signaling has been shown to regulate germ cell potency during mammalian testis development, where NODAL signaling is activated by signals (including FGF9) from somatic cells that lead to upregulation of the NODAL-co-receptor CRIPTO in germ cells [28] [29]. Furthermore, NODAL signaling regulates entry into meiosis [28] [30] [29]. Additionally, Spiller et al. found expression of *NODAL* and its cofactor *CRIPTO* as well as *LEFTY1* in CIS and ECs and NODAL signaling might also provide a mechanism regulating potency in GCCs [28] [31]. In human ESCs and in murine epiblast cells, NODAL signaling has been shown to contribute to maintenance of pluripotency and is a hallmark of the primed state of pluripotency [32] [32].

BMP family members transduce their signals via their downstream effectors ID1–3, thereby regulating embryonic developmental and differentiation processes [33] [34] [35]. Bmp signals (Bmp4 /8B) specify murine PGCs from early proximal epiblast cells by suppressing Wnt signaling response genes and promoting Prdm1 /14 expression via T [36] [37] [38]. Furthermore, Bmp signaling is important for murine PGC migration and survival, since reduced Bmp signaling within the genital ridge leads to reduced numbers of PGCs and disrupted migration [39]. A Zebrafish model carrying a mutation in an ortholog of the human BMPR1B develops a seminoma-like tumor [40] [41] [42]. Furthermore, BMP signaling activity distinguishes histological subsets of paediatric germ cell tumors [43] and expression of BMP effectors ID1–3 has been demonstrated in seminomas [44]. Thus, BMP signaling might also play an important role in GCC pathogenesis. In the murine system, Pereira et al. found that Bmp /Smad5 signaling contributes to negative regulation of Nodal, since Smad5-deficient amnion cells showed ectoptic activation of Nodal and its feedback loops [45]. In turn, Nodal was shown to act as a Bmp inhibitor by heterodimerizing with Bmps [46]. Thus, a reciprocal interaction between Nodal and Bmp signaling might be an important mechanism in germ cell development and GCC development.

### The microenvironment influences the cell fate of TCam-2 cells

In previous studies, we demonstrated that the seminoma cell line TCam-2 differentiates into a mixed non-seminoma, when being cultivated in murine embryonic fibroblast conditioned medium supplemented with FGF4 /Heparin or in a combination of FGF4 /TGF-B1 /EGF, which mimics a somatic microenvironment, [47]. During this process the morphology changes considerably from polygonal to very big, flat and round cells with a big nucleus. Furthermore, a network-like structure is build up, the amount of multinucleated giant cells increases strongly and the proliferation rate drops significantly. Pluripotency markers (NANOG, OCT3/4, Alkaline Phosphatase) are downregulated, while markers for somatic differentiation are upregulated (AFP, PAX6, HAND1, T, HOXB1). Interestingly, an EC-intermediate, indicated by upregulation of SOX2 or SOX17 downregulation is not detected. Additionally, the BMP /SMAD signaling is reduced, putatively leading to downregualtion of PRDM1, allowing for differentiation into a mixed non-seminoma.

In a further study, we demonstrated that TCam-2 cells presented as pure, undifferentiated ECs 6–8 weeks after xenografting into the murine flank or brain [47]. In these somatic microenvironments, TCam-2 cells upregulate EC-markers SOX2, CD30, DNMT3B/L and downregulate seminoma markers SOX17, cKIT and PRDM1. Furthermore, DNA methylation levels increased strongly [47] [48]. Using these experimental settings, development of teratomas has never been observed. In contrast, orthotopic injection of TCam-2 into the testis leads to CIS /seminoma-like growth within the seminiferous tubules, indicated by a CIS /seminoma-like morphology (uniformly growing big round cells with a big nucleus, weakly eosinophilic) and expression of typical markers like SOX17, BLIMP1, VASA, TFAP2C and cKIT. These previous studies suggest that the microenvironment affects the cell fate of seminomatous TCam-2 cells [48].

### 2102EP cells as an EC model

In this study, we took advantage of the xenotransplantation model to analyze the molecular mechanisms during the reprogramming of TCam-2 to an EC in the somatic microenvironment of the murine flank. We utilized the cell line 2102EP as an EC model, which has been widely used in different studies, ranging from analyzing differentiation abilities, DNA methylation and retinoic acid response to studying chemoresistance and pluripotency [49] [50] [51] [48] [52] [53] [54] [55] [56]. 2102EP cells were derived from a patient suffering from an EC /teratocarcinoma and show an EC-like morphology (small polygonal and flat cells). Furthermore, 2102EP cells express EC- and pluripotency markers like SOX2, CD30, DNMT3B /L, NANOG, OCT3/4, but lack expression of PGC /semimona markers like SOX17. Similar to ECs, 2102EP cells show cytoplasmic localization of PRDM1 [57] [48]. The DNA of ECs is hypermethylated compared to seminomas [6]. In line to this finding, the DNA of 2102EP cells is highly methylated compared to TCam-2 cells. A common feature of GCCs, the gain of chromosome 12p can also be found in 2102EP [53] [56]. After xenotransplantation into the murine testis, flank or brain, 2102EP cells show a typical morphology and gene expression profile of EC cells. Importantly, 2102EP cells are nullipotent, thus they do not tend to differentiate in vivo into teratoma-, yolk-sac tumor- or choriocarcinoma-like cells [58] [52]. So, 2102EP cells resemble an undifferentiated EC in vitro and in vivo, highlighting 2102EP as a valuable EC model.

In this study, we deciphered the molecular mechanisms involved in adaptation of seminomatous TCam-2 cells to an EC-like cell fate. We demonstrate that interference with the BMP signaling pathway leads to upregulation of NODAL signaling as well as pluripotency- and epigenetic reprogramming factors, which drive the reprogramming and epigenetic remodeling of TCam-2 cells during growth in the somatic microenvironment of the murine flank. Our data strongly suggest that seminomas can be reprogrammed to an EC upon interaction with the microenvironment /tumor stroma.

## Results

The seminoma-like cell line TCam-2 is able to develop into an EC-like state after being xenografted into the flank or brain of nude mice [7] [48]. In this study, we analyzed the kinetics of gene expression (Gex) and DNA methylation (5mC) during this seminoma to EC transition (SET) to gain insight into the mechanisms driving this transition. Our previous experiments demonstrated that 6 weeks after transplantation TCam-2 cells had adapted an EC-like state [48]. Thus, we xenografted TCam-2 and 2102EP into the flank of nude mice and analyzed 5mC and Gex levels using microarrays after 1, 2, 4 and 6 weeks to follow early and late events during the transition.

### Dynamics of 5mC and gene expression during in vivo growth of TCam-2

**Fig 1:**
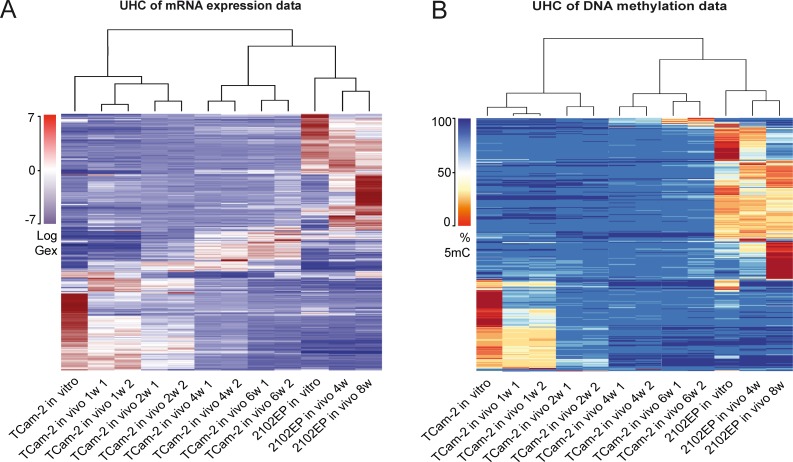
Global dynamics of Gex and 5mC during reprogramming of TCam-2 cells. (A—B) Unsupervised hierarchical clustering illustrates genome-wide changes in Gex (A) and 5mC (B) over time in indicated samples. Dendrograms indicate that during SET TCam-2 cells become more similar to the EC control cells.

Unsupervised hierarchical clustering (UHC) analysis of Gex and 5mC data revealed the differences between TCam-2 and 2102EP—after transplantation, the UHC demonstrated that up to 2 weeks after transplantation the cells still clustered to the parental TCam-2 cells. Gene expression seemed to gradually adjust to the 2102EP sample pattern, while DNA methylation seemed to increase and reorganized to the 2102EP pattern later (Fig 1A and 1B). After 4 weeks, xenografted TCam-2 cells clustered to the 2102EP cells, indicating an adaptation to an EC-like state with regard to Gex and 5mC (Fig 1A and 1B).

### Detailed analysis of Gex and 5mC dynamics during reprogramming of TCam-2

To gain a detailed insight into 5mC dynamics during SET, we plotted the averaged CpG-methylation at various regulatory regions (transcription start site (TSS)1500, TSS200, 5’-UTR, 1st exon, gene body and 3’-UTR) across all genes found to be differentially methylated in TCam-2 cells in vitro, after 1, 2, 4 and 6 weeks of in vivo growth as well as in 2102EP (S1 Fig). Parental and xenografted TCam-2 cells display 5mC levels of about 40–50% at the TSS1500, TSS200 and 1^st^ exon. In contrast, 5mC levels at the 5’-UTR (≤ 40%), the gene body (≤ 35%) and the 3’-UTR (≤ 35%) are low in in vitro cultivated TCam-2 and xenografted cells for 1 week, while 5mC levels steadily increase at these regions with progressive in vivo growth to an profile highly comparable to 2102EP cells (S1 Fig).

**Fig 2:**
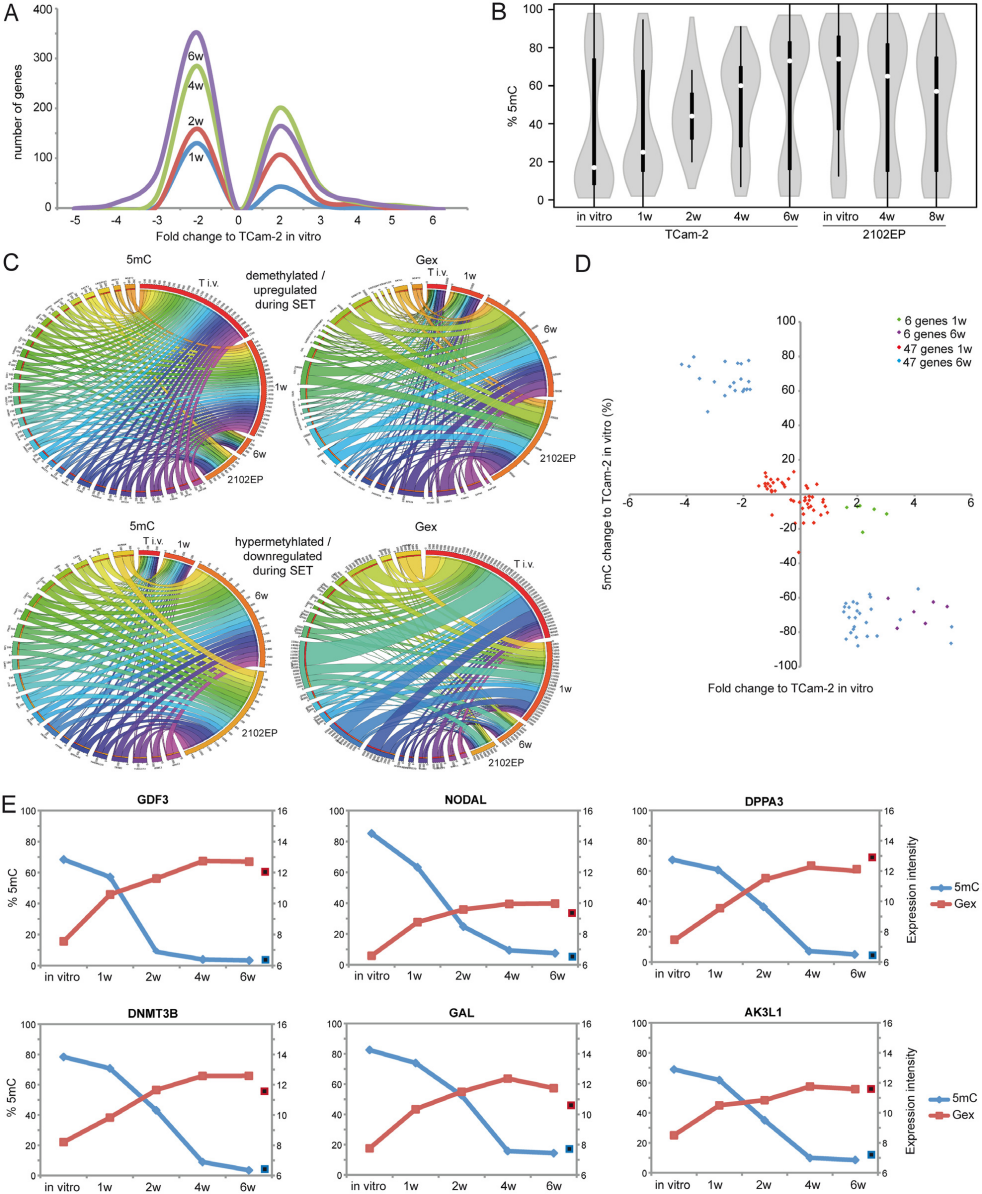
Detailed analyses of Gex and 5mC dynamics during reprogramming of TCam-2 cells. (A) Histogram showing changes in Gex 1–6 weeks after xenografting compared to TCam-2 in vitro. (B) Violin plot illustrating 5mC level distribution of all differentially methylated genes during SET. (C) Circos diagrams illustrate inverse correlation between Gex and 5mC during SET and in comparison to 2102EP cells in the 53 5mC /Gex-group genes. Gex and 5mC data of each analyzed gene is linked to each analyzed sample—the thicker a connection the higher the Gex /5mC level and vice versa. Thus, genes with high 5mC levels in a certain sample (thick connection) show a small connection in the illustration of Gex data. T i.v. = TCam-2 in vitro, 1w = TCam-2 in vivo 1w, 6w = TCam-2 in vivo 6w, 2102EP = 2102EP in vitro. (D) Volcano plot of Gex and 5mC data of the 5mC /Gex-group 1 and 6 weeks after xenografting. (E) Gex and 5mC dynamis of indicated genes during SET. Red framed black squares = Gex, blue framed black squares = 5mC of averaged data of 2102EP in vivo 4 /8 weeks.

Next, we distinguished CpG-island-associated DNA methylation events from DNA methylation at open sea context (i. e. non-CpG-island context) (Fig 2A and 2B). In TCam-2, the vast majority of CpG-island-associated CpGs show low levels of DNA methylation in regions 1500 and 200 bp upstream of the TSS, the 5’-UTR and the 1^st^ exon (orange circle in S2A Fig), while CpG-islands within the gene body and the 3’-UTR appear medium to hypermethylated (green circle in S2A Fig). Six weeks after xenografting the CpGs in the gene body display distinct changes in methylation (red circle in S2A Fig) and demethylation (yellow circle in S2A Fig), while probes within the TSS200 /1500, 5’-UTR and 1^st^ exon remain hypomethylated or become demethylated (black circle in S2A Fig).

CpG-probes associated with the open sea show a higher methylation compared to CpG-islands (S2B Fig). The 5mC levels of these regions are dramatically altered after six weeks in vitro and in 2102EP (S2B Fig). Thus, during in vivo growth of TCam-2 the change of methylation mainly occurs in within gene bodies of CpG-islands and in the open sea, while methylation of CpG-island-associated TSSs remains nearly unchanged.

Following xenografting of TCam-2, numbers of medium (41–80%) and highly (> 81%) methylated CpGs showed a strong increase on all chromosomes, except chromosomes Y and 19 (S1A Data). Strongly reduced methylation on the Y chromosome can be explained by the fact that complete arms of this chromosome are deleted TCam-2 (purple arrow in S1A Data) [56]. On chromosome 19, however, high numbers of hypomethylated CpGs (0–40%) are maintained 6 weeks after xenografting (green arrow in S1A Data). Thus, chromosome 19 seems to escape the de novo DNA methylation process during the SET. 6 weeks after xenografting 5mC distribution across all chromosomes is more comparable to 2102EP cells, while parental TCam-2 and TCam-2 xenografted for 1 week show considerable differences in 5mC distribution compared to 2102EP (S3A Fig).

To define initiating events of this reprogramming, we analyzed early Gex and 5mC dynamics using a volcano plot and found an (almost linear) increase in the number of genes being deregulated in expression during in vivo growth over time (Fig 2A). Next, a violin plot was used to visualize 5mC level distribution across all differentially methylated CpGs during SET (Fig 2B). TCam-2 cells cultivated in vitro and for 1 week in vivo display a high number of hypomethylated CpGs (≤ 30%). Interestingly, 2 weeks after xenografting the majority of CpGs displays 5mC levels around 50%, indicative for intermediate methylation. 4–6 weeks after xenografting, TCam-2 cells peak at approximately 60 and 70% 5mC levels respectively, indicating that the majority of CpGs are hypermethylated. These data demonstrate that 5mC levels shift from hypomethylation at one week via intermediate methylation at two weeks gradually towards high levels seen at 4–6 weeks. This strongly suggests that the remodeling is a gradual and constant process.

**Fig 3:**
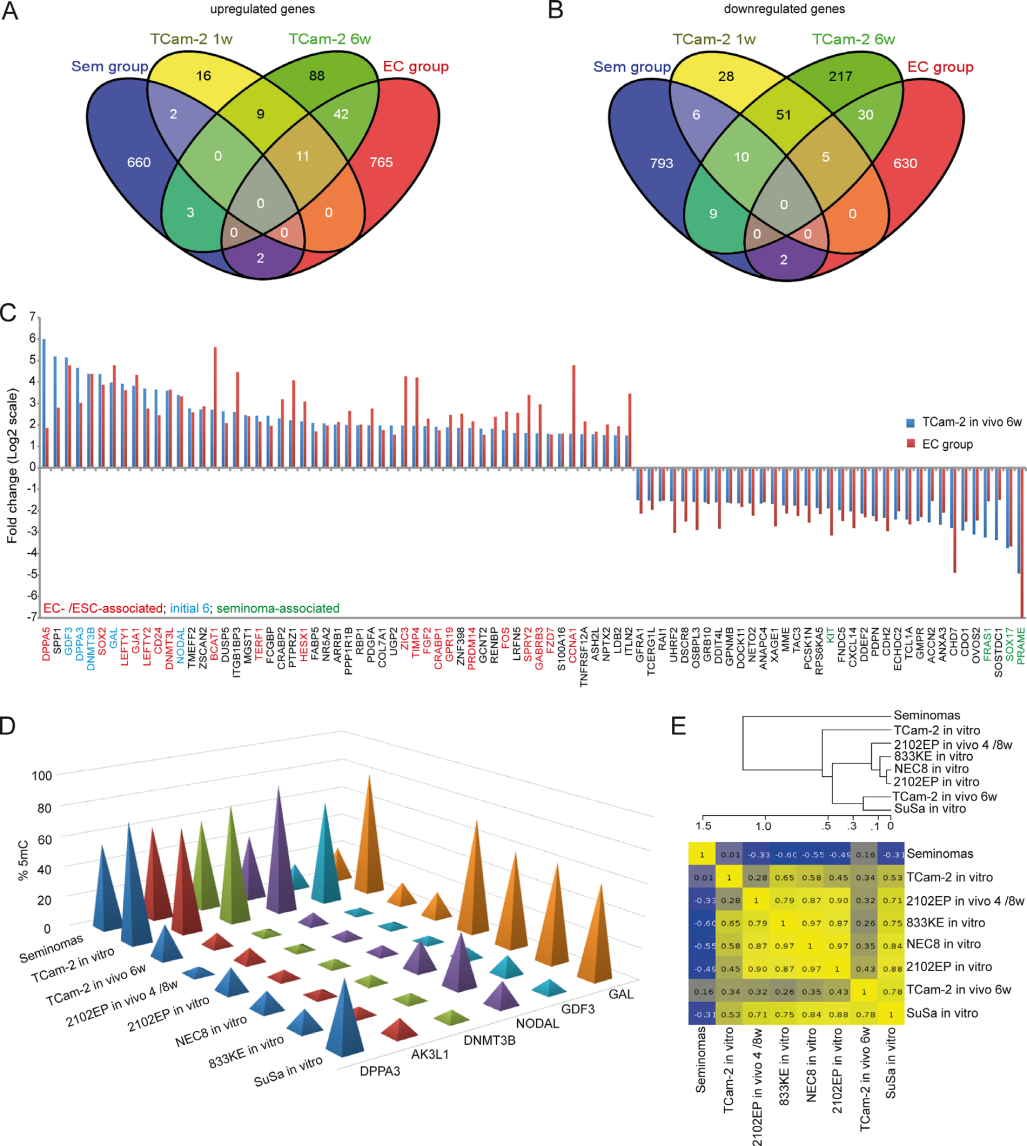
Meta-analysis of Gex and 5mC data of xenografted TCam-2 and GCC tissues. (A, B) Venn diagrams summarizing commonly up- (A) and downregulated (B) genes between seminoma, TCam-2 xenografted for 1 and 6 weeks and ECs. Corresponding data is given in (E and H in S1 Data). Genes recorded in duplicates (due to multiple probes on the array) were included only once. (C) Expression intensities of indicated genes in TCam-2 xenografted for 6 weeks and the EC group as fold change versus appropriate controls (TCam-2 in vitro /seminoma tissues). Genes were categorized (color coded) based on [48] [18] [35]. (D) 5mC levels of indicated genes in seminomas, parental and xenografted TCam-2 as well as EC cell lines (2102EP, NEC8, 833KE, SuSa) as found by 450k microarray analysis. (E) BDCP analysis of 5mC data of genes and samples indicated in (D).

Now, we wanted to understand whether the changes in 5mC correlate to changes in Gex. A Pearson’s correlation of the microarray data identified 601 genes, showing inverse correlation between 5mC and Gex (S1B Data). A BDPC methylation cluster analysis of these 601 genes demonstrates that the transplanted cells cluster to the parental TCam-2 cell line up to 2 weeks after transplantation. Thereafter, they cluster more to the 2102EP to become highly similar after 6 weeks after transplantation (Fig 3B).

We reasoned that the genes from the differentially methylated group displaying the highest change in expression during the SET might be the candidates for driving this process. Hence, from the 601 differentially methylated genes we excluded all genes with an expression fold change of <log_2_1.5 versus parental TCam-2. We found 53 genes which passed this criteria and called them 5mC /Gex-group (S1C Data). Genes that are weakly expressed /hypermethylated in TCam-2 cultivated in vitro (Fig 2C, (upper panel: T i.v.)) and for 1 week in vivo (1w) become demethylated and upregulated 6 weeks after xenografting (6w) and are expressed and hypomethylated in 2102EP cells (2102EP). Vice versa, genes hypomethylated and expressed in TCam-2 in vitro (Fig 2C, lower panel: T i.v.) and grown in vivo for 1 week (1w) become /are hypermethylated and downregulated in TCam-2 xenografted for 6 weeks (6w) and 2102EP cells (2102EP), respectively. Thus, 6 weeks after xenografting, the 5mC and Gex status of these genes is more comparable to the 2102EP profile than to parental TCam-2 or TCam-2 xenografted for 1 week (Fig 2C).

Next, we compared the genes of the 5mC /Gex-group to a set of genes deregulated in expression after 1 (143 genes) and 6 weeks (503 genes) (D and E in S1 Data). This revealed that 6 genes of the 5mC /Gex-group were upregulated after 1 week, despite the fact that 5mC-levels had dropped only marginally (Fig 2D and S1F Data). After 6 weeks, expression of these genes had increased further and the genomic loci became hypomethylated (Fig 2D). These 6 genes are *GDF3*, *NODAL*, *DPPA3*, *DNMT3B*, *GAL* and *AK3L1*, which are pluripotency-associated genes, except *AK3L1*, which encodes an enzyme of the adenylate kinase family (Fig 2E) [59] [60]. After 6 weeks, the remaining 47 genes of the 5mC /Gex group were deregulated and showed inverse correlation to 5mC (S1C Data). From them, EC-, pluripotency- and reprogramming-associated genes *REX1 (ZFP42)*, *DND1*, *JARID2* and *PRDM14* were hypomethylated and upregulated, while seminoma-related genes *PRDM1*, *PROM1* and *IGF1* became hypermethylated and were downregulated [61] [62].

Furthermore, additional EC and pluripotency genes were upregulated (*SOX2*, *LEFTY1 /2*, *DNMT3L*, *SALL4*, *DPPA5*, *BCAT1*, *FZD7*, *LIN28*, *ZIC3*), while seminoma-associated genes where downregulated (*SOX17*, *TFAP2C*, *cKIT*, *PRAME*), without changing 5mC-levels (D and E in S1 Data) [3] [17] [5].

We verified selected alterations in Gex 1–2 weeks after xenografting by qRT-PCR and immunohistochemical staining (IHC) (A and B in S4 Fig). Additionally, we confirmed demethylation of the *GDF3* locus in TCam-2 4 weeks after xenografting by sodium-bisfulfite-sequencing (S4C Fig).

The heatmap of Gex data (Fig 1A) demonstrated that aside from the similarities between xenografted TCam-2 and the 2102EP samples, there are also differences in Gex between the analyzed cell types during SET. We normalized Gex data of all 2102EP samples (in vitro, 4w, 8w) and TCam-2 cells xenografted for 6w versus TCam-2 in vitro (S1G Data). Next, we excluded all genes deregulated in both, the TCam-2 6w and the 2102EP samples to produce datasets containing genes exclusively expressed in TCam-2 6w, but not in 2102EP samples and vice versa (S1G Data). We performed a STRING-based protein-protein interaction as well as a GeneTrail-based Gene Ontology (GO) analysis of these data sets to show in which molecular processes as well as interactive networks these genes are involved and summarized the results in (S1G Data). Genes exclusively expressed in the TCam-2 6w samples are mainly linked to developmental and regulatory processes as well as signaling, while genes expressed only in 2102EP samples are related to GO categories linked to cellular compartments, like cytoplasm, nucleus, membrane and intracellular organelles.

### Deciphering the mechanisms driving reprogramming of TCam-2

To further analyze the regulatory mechanisms underlying the SET, we performed a STRING-protein-interaction-analysis of all genes upregulated after 1 week. An interaction network between 4 of the 6 5mC /Gex-group genes (GDF3, GAL, DPPA3, DNMT3B) and SOX2 as well as DNMT3L was predicted (S5A Fig). Further, a regulatory link between NODAL and LEFTY2 was proposed (S5A Fig). Inclusion of all genes upregulated lately after 6 weeks led to extension of this network to many pluripotency- and reprogramming-related factors, like REX1, JARID2, FGF2, WNT3, ZIC3 and PRDM14 (S5B Fig).

### Changes of 5mC and gene expression during programming of TCam-2 reflect differences between seminomas and ECs

We asked, whether the changes in Gex reflect differences between seminomas and ECs in vivo. We performed a meta-analysis of our data and a cDNA microarray of GCCs [15] and filtered genes that are informative to discriminate seminomas from ECs (S1H Data). With progressive in vivo growth, TCam-2 cells express more genes found in ECs than seminomas (Fig 3A and 3B). Among them, *GDF3*, *NODAL*, *LEFTY1 /2*, *GAL*, *DPPA3*, *SOX2*, *DNMT3B /L*, *DPPA5*, *BCAT1*, *FGF2*, *PRDM14*, *ZIC3* and *FZD7*, while seminoma markers *SOX17*, *cKIT* and *PRAME* were downregulated (Fig 3C). Furthermore, a qRT-PCR analysis verified that pluripotency and epigenetic reprogramming factors (*SOX2*, *ZIC3*, *GDF3*, *NODAL*, *DPPA3*, *DNMT3B*, *GAL*, *JARID2*, *REX1*, *WNT3*, *PRDM14*) are expressed higher on average in EC cell lines (2102EP, NCCIT, NT2/D1, 833KE, GCT27, H12) than in parental TCam-2, while expression of seminoma markers SOX17 and TFAP2C is considerably higher in TCam-2 (S5C Fig). In line with previous publications, expression of pluripotency factors NANOG and OCT3/4 is high is all cell lines analyzed [4] [59] [63]. Thus, the genes upregulated during the SET represent EC core genes.

During reprogramming, we found decreasing 5mC-levels in 6 5mC /Gex-group genes (Fig 2E). We compared 450k array data of these genes in parental and xenografted TCam-2 /2102EP cells to seminoma tissues and three additional EC cell lines (NEC8, 833KE, SuSa [64] [65] [66]) (Fig 3D). We found that *DPPA3*, *AK3L1*, *DNMT3B* and *NODAL* are hypermethylated at analyzed loci in seminomas and parental TCam-2 compared to TCam-2 in vivo 6w and the EC samples (Fig 3D). Parental TCam-2 and all EC cell lines show *GAL* hypermethylation, which is strongly reduced after xenografting of TCam-2 and 2102EP, suggesting that *GAL* hypermethylation is established and maintained during in vitro cultivation of EC cells. *GDF3* hypermethylation is restricted to parental TCam-2, but not seen in seminomas or EC cells (Fig 3D). The *GDF3* locus became demethylated during xenografting of TCam-2 (Fig 3D). Thus, high 5mC-levels of *GDF3* in parental TCam-2 point at a cell line-specific effect, but correlate inversely to Gex (S1D Data). A BDCP analysis demonstrates that parental TCam-2 cells cluster closely to seminoma tissues and align to the EC samples 6 weeks after xenografting with regard to 5mC status of analyzed genes (Fig 3E).

### BMP interference leads to induction of NODAL signaling

We interrogated our data with regard to expression of signaling pathway-related genes to further elucidate the initial trigger of the SET. We found deregulation of genes involved in BMP, NODAL, Retinoic acid (RA), FGF, HIPPO, STAT, IGF, NOTCH and WNT signaling (S6A Fig).

**Fig 4:**
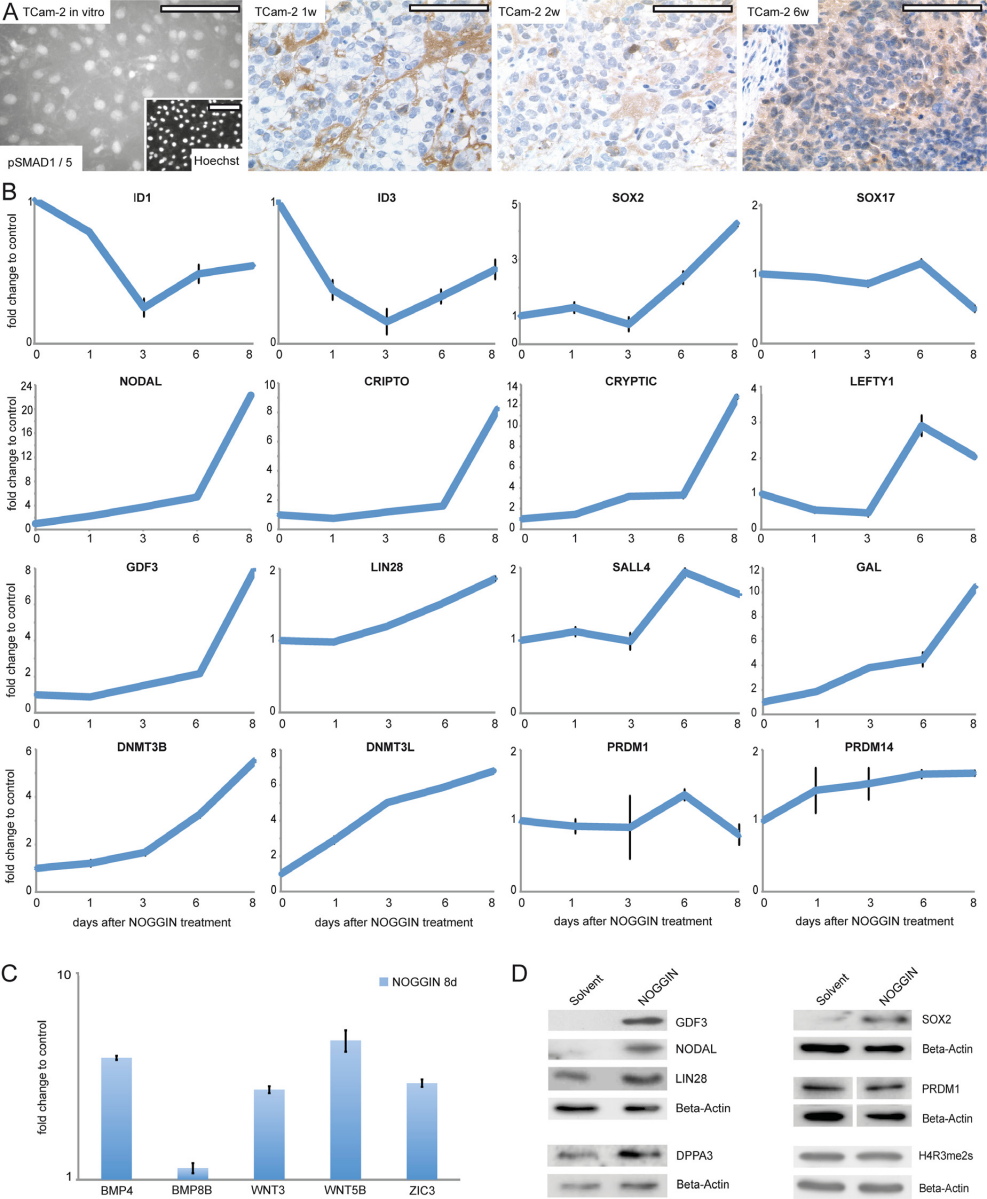
Inhibition of BMP signaling is crucial for initiation of reprogramming. (A) IF /IHC staining of pSMAD 1 /5 in parental and xenografted TCam-2 at indicated time points. Scale bar: 100 μm. (B, C) qRT-PCR analysis of indicated genes after NOGGIN treatment of TCam-2 cells for 1–8 days. (D) Western blotting of indicated proteins 8 days after NOGGIN treatment of TCam-2 cells.

Since BMP signaling is central for germ cell specification, we concentrated on this pathway first. In parental TCam-2, moderate cytoplasmic and strong nuclear staining of phosphorylated SMAD (pSMAD)1 /5 was detected by immunofluorescence staining (IF) (Fig 4A). Also, expression of *BMPR1A /R2*, *BMP4 /7* and *SMAD4* suggested that BMP signaling is active (S1I Data). 1 week after xenografting, a rapid and strong decrease of the BMP pathway effectors *ID1 /3* is detected (S6A Fig). Additionally, BMP receptors *BMPR1A /R2* show a trend of downregulation (S1I Data). IHC and western blotting detected loss of SMAD1 /5 phosphorylation from 1–4 weeks after xenografting (Fig 4A and S6B Fig). Interestingly, after 6 weeks, cytoplasmic pSMAD1 /5 is detectable again, pointing at recovery of BMP signaling (Fig 4A). Also, *ID1* /*3* and *BMPR1A* expression recovers from 2 weeks on (S6A Fig and S1I Data).

To test the role of BMP signaling for initiation of SET-reprogramming, TCam-2 cells were treated with the BMP inhibitor NOGGIN for 8 days [67]. After application of NOGGIN, by western blotting and qRT-PCR analysis, we observed a reduction of pSMAD1 /5 levels (S6C Fig) and downregulation of *ID1* /*3* (Fig 4B), indicating inhibition of the BMP-pathway. Further, upregulation of 4 of the 5mC /Gex-group genes (*NODAL*, *GDF3*, *GAL*, *DNMT3B)* as well as the EC markers *CRIPTO*, *CRYPTIC*, *LEFTY1*, *SALL4*, *LIN28*, *JARID2*, *PRDM14*, *DNMT3L* and *SOX2* was detected, while SOX17 was downregulated after 8 days (Fig 4B). Additionally, BMP- and WNT signaling-associated molecules *BMP4*, *WNT3* and *WNT5B* as well as pluripoteny-related gene *ZIC3* were upregulated 8 days after NOGGIN treatment (Fig 4C). Upregulation of GDF3, NODAL, LIN28, SOX2 and DPPA3 was shown by western blotting (Fig 4D). To confirm the results, we treated TCam-2 cells with the BMP inhibitor LDN193189. Again, we observed a reduction in SMAD1 /5-phosphorylation, downregulation of *ID1 /3* as well as *NODAL*, *SOX2*, *LIN28* and *DNMT3B /L* upregulation (D and E in S6 Fig). These findings suggest that BMP inhibition is an initial event in the reprogramming of seminomas to ECs. Inhibition of BMP signaling leads to derepression of NODAL signaling as well as upregulation of pluripotency- and reprogramming-associated factors.

To analyze if activation of NODAL signaling alone is sufficient to induce deregulation of pluripotency- and SET-associated genes, we treated TCam-2 cells with recombinant NODAL. Expression of endogenous *NODAL* and corresponding signaling keyplayers or pluripotency- and reprogramming-associated factors did not change, although increasing SMAD2 /3-phosphorylation verified an efficient treatment (F and G in S6 Fig). This suggests that inhibition of BMP signaling is a prerequisite for the establishment of NODAL signaling.

**Fig 5:**
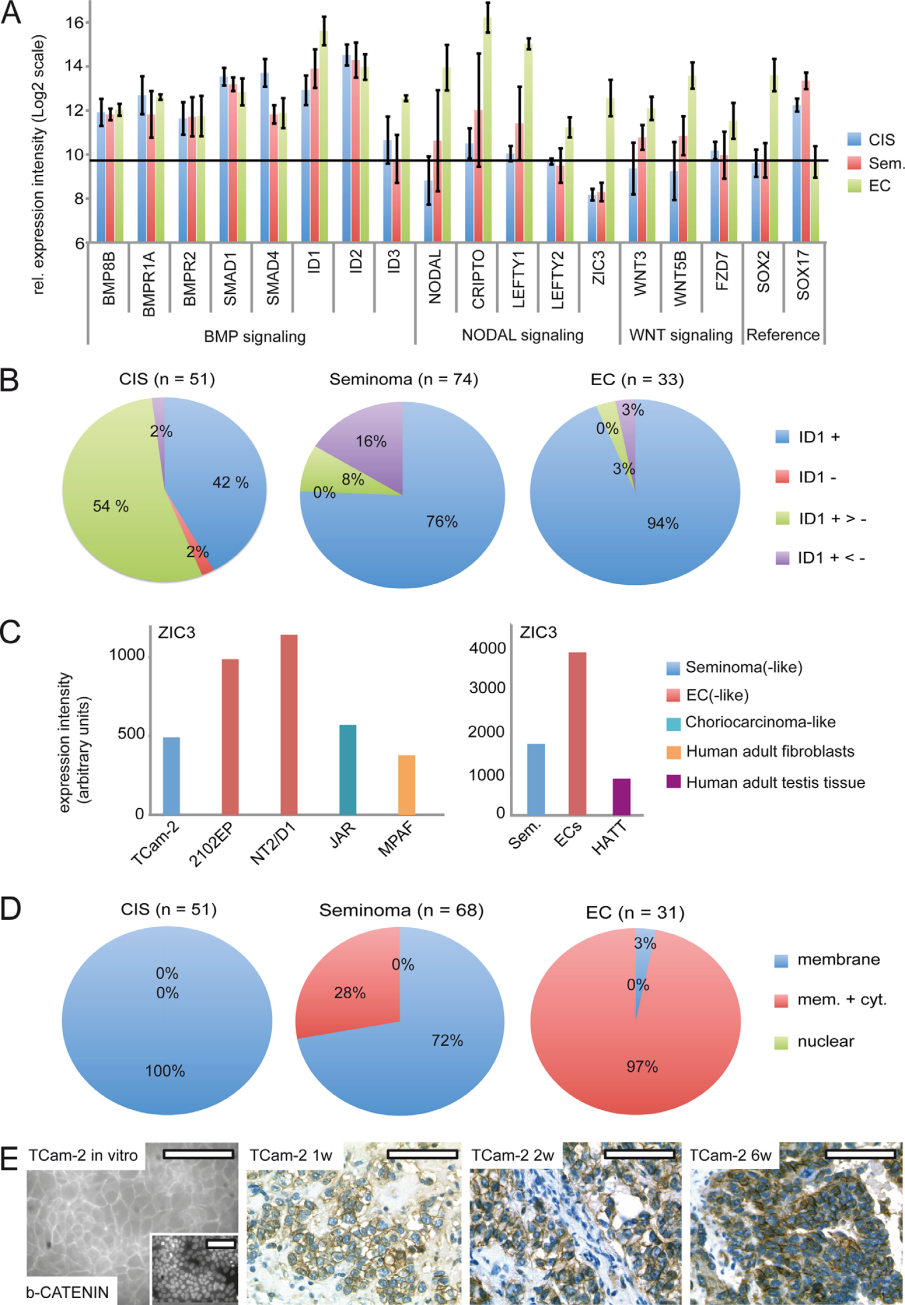
BMP, NODAL and WNT signaling in GCC tissues and cell lines. (A) cDNA microarray expression data of BMP, NODAL and WNT signaling-associated genes in GCC tissues. *SOX2* and *SOX17* were used to determine zero level of expression intensity (black line). (B) Pie diagrams summarizing ID1 IHC data of GCC-TMAs. Stainings were classified as ID1 positive (+), negative (-), mixed with much more positive than negative cells (+ >-) and mixed with much more negative than positive cells (+ <-). (C) ZIC3 protein levels in indicated GCC cell lines and tissues. (D) Pie diagrams summarizing beta-CATENIN IHC data of GCC-TMAs. Beta-CATENIN staining was classified as membraneous (m), cytoplasmic (c) and nuclear (n). (E) IF /IHC staining of beta-CATENIN in in parental and xenografted TCam-2 cells (1, 2 and 6 weeks). Scale bar: 100 μm.

Next, we screened GCC tissues for expression of BMP and NODAL signaling keyplayers by re-analyzing cDNA microarray data and performing IHC as well as western blots (Fig 5A–5C) [15]. For IHC, only TFAP2C positive and SOX2 negative CIS and semiomas as well as SOX2 positive ECs were analyzed (S7A Fig). In CIS, seminomas and ECs, expression of *BMP8B*, *BMPR1A /2*, *SMAD1 /4* and *ID1 /2* was detected, while *ID3* expression was restricted to ECs (Fig 5A). In line with this expression profile, ID1 was detectable in the vast majority of CIS, seminomas and ECs by IHC of GCC tissue microarrays (GCC-TMA), showing that in these GCC entities BMP signaling is active (Fig 5B and S7A Fig).

NODAL signaling induces its downstream effectors *CRIPTO /CRYPTIC* and *LEFTY1 /2*. Furthermore, NODAL signaling activity is maintained by *ZIC3*. We detected considerably higher levels of these NODAL signaling keyplayers in ECs compared to CIS /seminomas (Fig 5A). Additionally, a western blot analysis demonstrates the EC cell lines 2102EP and NT2/D1 display high levels of ZIC3 while TCam-2 cells, choriocarcinoma-like JAR cells and human adult fibroblasts show low levels (S7B Fig). Furthermore, expression of ZIC3 is higher in EC tissues than in seminomas, CIS or normal testis tissue (Fig 5C and S7C Fig). Taken together, in vivo *ZIC3* mRNA and ZIC3 protein levels correlate to SOX2 as well as NODAL expression (ECs) and correlate inversely to SOX17 (CIS, semiomas) (S7C Fig).

NODAL and ACTIVIN signaling are closely related to each other and key components of ACTIVIN signaling are heterogeneously expressed in GCCs [68] [69]. Additionally, CRIPTO is able to inhibit ACTIVIN signaling [70]. Thus, during SET activation of NODAL signaling might also influence ACTIVIN signaling. We screened for expression of ACTIVIN signaling keyplayers in TCam-2 in vitro and in vivo, but could not detect any changes in expression of the ACTIVIN /INHIBINS, the ACTIVIN receptors (ACVRs), TGFBR3, MAN1 or the ACTIVIN inhibitor Follistatin (FST) (S1J Data) [68] [69]. Thus, ACTIVIN signaling seems not to contribute to reprogramming of TCam-2 cells.

In vitro, TCam-2 cells display negligible expression of WNT molecules and only expression of WNT receptors *FZD3* /*6* was detected (S1K Data). In contrast, in 2102EP cells *WNT3*/*5B* and *FZD7* /*9* are expressed. During SET, *WNT3/5B* and *FZD7* /*9* are induced, while *FZD3* /*6* tend to be downregulated (S7A Fig and S1K Data). Thus, TCam-2 cells change expression of WNT signaling associated genes to a profile comparable to 2102EP. Accordingly, *WNT3* /*5B* and *FZD7* expression is higher in EC tissues than in CIS /seminomas (Fig 5A). Using IHC, we demonstrate that CIS display only membraneous staining of the canonical WNT effector beta-CATENIN, while seminomas and ECs presented in two states, i. e. showing membraneous staining or positive at both, the membrane and the cytoplasm (Fig 5D and S6A Fig). 72% of seminomas stained positive at the membrane only, while 97% of ECs displayed both, strong membraneous and cytoplasmic beta-CATENIN, verifying results of Korkola et al. [71] (Fig 5D and S7A Fig). In line with these data, IF /IHC demonstrates that in parental TCam-2 beta-CATENIN is localized to the membrane, while increasing cytoplasmic staining is detectable 1–6 weeks after xenografting (Fig 5E). In conclusion, similar to EC tissues, beta-CATENIN accumulates in the cytoplasm of TCam-2 cells following xenografting.

## Discussion

In this study, we analyzed the epigenetic and molecular mechanisms underlying the seminoma to EC reprogramming process. After transplantation, expression of 6 genes was rapidly induced, with 5mC levels unchanged initially. Thus, in seminomas these genes seem to be poised for expression. Early induction of DNMT3B initiates a wave of de novo DNA methylation causing a gradual remodeling of the methylome two weeks after xenografting, leading to a genome-wide high 5mC levels similar to an EC. During SET, remodeling of the methylome affects mainly gene bodies (but not regulatory regions, like TSS) in the CpG-island and non-CpG-island context and follows deregulation in Gex, suggesting that DNA methylation rather reinforces than initiates the EC-like state of TCam-2.

### The role of BMP- and NODAL signaling during reprogramming of TCam-2

A strong downregulation of the BMP signaling downstream effectors *ID1* and *ID3* during the SET prompted us to investigate BMP signaling in more detail. We show that inhibition of BMP signaling leads to induction of NODAL signaling and pluripotency- as well as epigenetic reprogramming factors comparable to the reprogramming of TCam-2 in vivo. Previously, we were able to show that during in vitro differentiation of TCam-2 cells into a mixed non-seminoma the activity of BMP signaling-related SMAD1 /5 /8 molecules was reduced [47]. This further demonstrates that high BMP signaling activity is associated with a CIS /seminoma-like character, while low levels are linked to a non-seminomatous cell fate. Hence, we propose that inhibition of BMP signaling is the initial event triggering SET-reprogramming. In contrast to the results reported here, upregulation of the EC-marker SOX2 was not observed during the in vitro differentiation [47]. We speculate that the particular experimental settings in vitro (supplementation with FGF4, TGF-B1, EGF) resulted in a persistent suppression of SOX2, leading to continuation of SOX17 expression. However, with downregulation of PGC- (PRDM1, TFAP2C, cKIT) and pluripotency (NANOG, OCT3/4, LIN28) marker genes, persisting SOX17 expression together with activation of the Hippo pathway resulted in differentiation into a mixed non-seminoma with predominant choriocarcinoma-like components [47].

Spiller et al. found expression of *NODAL* and its cofactor *CRIPTO* as well as *LEFTY1* in CIS and ECs [39]. The authors utilized qRT-PCR to analyze expression of NODAL signaling keyplayers in testis containing up to 90% CIS cells and non-seminomas, while seminomas were not included [28]. We detected low expression levels of NODAL signaling factors in CIS /seminomas and high levels in ECs (Fig 5A) [16]. In our study, the cDNA microarray analysis of GCC tissues was performed on RNA isolated from pure micro-dissected CIS cells, without any normal testicular tubules or invasive tumors, pure classical seminomas and ECs [35]. In our case, RNA expression levels and protein detection via IHC of various markers in GCC tissues is also observed in our SET model system. Hence, we argue that the discrepancies with Spiller et al. might be of technical nature, i. e. residual somatic components, which eventually skews analyses by having active NODAL signaling.

Spiller et al. state further that active NODAL signaling provides a mechanism regulating potency in GCCs [28] [31]. In human ESCs and in murine epiblast cells, NODAL signaling has been shown to contribute to maintenance of pluripotency and is a hallmark of the primed state of pluripotency [32]. Thus, activation of NODAL signaling might trigger the shift from latent pluripotency (observed in seminomas) to primed pluripotency displayed by ECs.

During vertebrate development, expression of the pluripotency-related factor *ZIC3* is repressed by BMP signaling and can be restored by NOGGIN-mediated inhibition of BMP signaling [72] [73] (Fig 4C). *ZIC3*, which is necessary for maintenance of NODAL signaling is highly expressed in ECs /xenografted TCam-2 and low in seminomas /TCam-2 in vitro (Fig 5A and S7B and S7C Fig; S1H Data) [17] [74]. The STRING analyses suggested that ZIC3 interacts with NODAL and LEFTY1 /2 (S5B Fig) and ZIC3 is activated by NANOG, OCT3/4 and SOX2 [17]. So, during SET, inhibition of BMP signaling leads to derepression of *SOX2*, restoring the classical pluripotency circuitry found in ECs and ESCs, subsequently leading to upregulation of *ZIC3*, which in turn helps to maintain NODAL signaling [17] [74].

### A crosstalk between BMP- and NODAL signaling controls initiation and progression of the transition

What is the crossregulation between BMP- and NODAL signaling? Pereira et al. found that in mice Bmp /Smad5 signaling represses Nodal, since amnion cells deficient for Smad5 showed ectoptic activation of Nodal and its feedback loops [45]. In turn, NODAL inhibits BMP by heterodimerizing with BMPs [46]. Thus in our case, signals form the tumor stroma inhibit BMP, which leads to derepression of NODAL. Upregulation of NODAL leads to establishment of an autoregulatory loop, including *LEFTY1 /2*, *CRIPTO /CRYPTIC* and *ZIC3*. This results in a cell intrinsic repression BMP signaling.

Why does BMP signaling recover during the reprogramming of TCam-2? As described above, Nodal activates its autoregulatory loop, which has been denominated the fast acting loop [24]. In addition, over time, the so called slow feedback loop activates Bmp4, which re-establishes BMP signaling and results in upregulation of Wnt3 and Fgf4 /Fgf8 [45] [75]. This is in agreement with the data from our transplantation studies, where we detected increased *WNT3* /*5B*, *BMP4* /*BMP7* and *FGF2* /*19* expression from 2–6 weeks after xenografting (S6A Fig; I and K in S1 Data). Additionally, 8 days after NOGGIN-treatment of TCam-2 cells *BMP4*, *WNT3* and *WNT5B* were upregulated (Fig 4C), while *ID1 /3* levels recovered like during in vivo growth (Fig 4B and S6A Fig).

### SOX2 and SOX17 in regulation of pluripotency and WNT signaling

During SET and after NOGGIN treatment, we detected downregulation of *SOX17* and upregulation of *SOX2*. In fact, *SOX17* expression is restricted to CIS and seminomas, while *SOX2* is highly expressed in ECs [2]. SOX17 has been identified as a key factor for specification of human PGCs and regulator of PRDM1 [14]. Thus, downregulation of SOX17 during the SET indicates loss of a PGC-like character. In mice, Sox2 complexes with Oct3/4 and binds to a canonical motif, thereby driving the expression of pluripotency genes [76]. Overexpression of Sox17 is able to replace Sox2 in the complex with Oct3/4, leading to a change in target site selection to a compressed binding motif [76]. So, we speculate that during SET the strong increase in SOX2 protein levels force partnering with OCT3/4, which leads to a switch to promoters encoding for the canonical motif found in pluripotency genes. Further, it is known that SOX17 antagonizes WNT signaling activity, which has been suggested to be low in seminomas and high in ECs [18] [19] [20]. So, downregulation of SOX17 could explain the de-repression of *WNT3* /*5B* during SET. The upregulated WNT3 results in cytoplasmic beta-CATENIN accumulation, but nuclear exclusion of beta-CATENIN suggests that the canonical WNT-pathway is not activated [77] [78] [79]. Thus, WNT3 /WNT5B most likely act in a non-canonical manner during SET [79].

### Model of the mechanisms and events driving reprogramming of TCam-2 to an EC-like state

**Fig 6:**
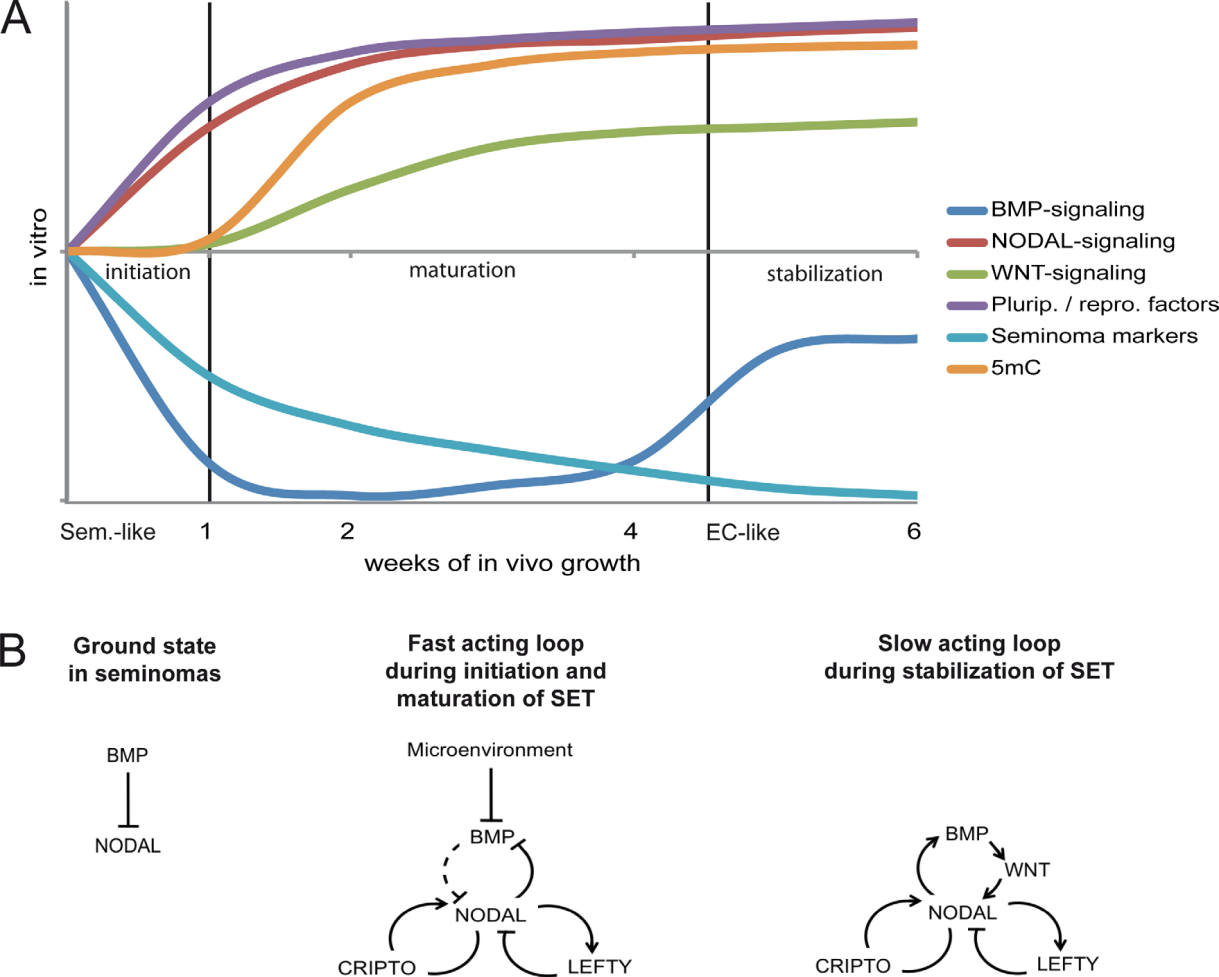
Model of the dynamics and molecular mechanisms during the SET. (A) Model summarizing the dynamics and events driving acquisition of pluripotency and epigenetic reprogramming of TCam-2 cells to an EC-like cell fate. (B) Models of the fast and slow acting NODAL feedback loop. Arrows indicate 'activation', T-shaped arrows indicate 'inhibition'.

Based on our findings, we propose a model in which the SET-reprogramming of xenografted TCam-2 is divided in three stages (initiation, maturation, stabilization) (Fig 6A) [80] [81]. The reprogramming is initiated by exogenous inhibition of BMP signaling causing rapid activation of NODAL. NODAL signaling establishes a fast acting autoregulatory loop (Fig 6B), leading to stimulation (CRIPTO /CRYPTIC) and limitation (LEFTY1 /2) of NODAL signaling and cell intrinsic suppression of BMP signaling. During this time, markers of pluripotency and reprogramming become upregulated and induction of *DNMT3B* initiates epigenetic remodeling. This phase we name the maturation phase. Thereafter, the slow acting NODAL feedback loop re-establishes BMP signaling to a level lower than in parental TCam-2, resulting in a balance between BMP and NODAL signaling and reinforcement of the acquired EC-like cell fate (the stabilization phase).

### Summary of this study

In summary, we demonstrated that in seminomas a set of 6 genes is rapidly induced after transplantation. These factors induce epigenetic remodelling of the genome and establish expression of the pluripotency network, leading to reprogramming into an EC. Further analysis revealed that interference with BMP is sufficient to induce these genes. We propose that BMP inhibition initiates the SET. The inhibition of BMP signaling, so we speculate, is initiated by factors like NOGGIN, which are expressed abundantly by the somatic microenvironment. So, upon transplantation into the flank, TCam-2 cells become exposed to BMP-inhibitors leading to initiation of SET. Corollary to this, a CIS or seminoma, which is exposed to BMP inhibitors by penetrating the testis confines during progressive growth, could be reprogrammed to an EC. Our data strongly suggest that GCC development is a plastic process that allows seminomas to progress into EC and maybe vice versa, depending on the signals from the tumor stroma. Therefore, seminoma patients might also develop an EC component during invasive tumor growth. ECs grow more aggressive than seminomas and need alternative treatment strategies, which requires adjustment of the therapy concept. The question remains, whether ECs might transit into a seminoma upon interference with the DNA methylation machinery or reprogramming key molecules identified in this study.

## Material and Methods

### Ethics statement

The ethics committee of the Rheinische Friedrich-Wilhelms-Universität Bonn approved the analyses of formalin fixed, paraffin-embedded type II GCC tissues in context of this study. No personal patient data will be collected or stored. Written permission to use the tissue for scientific purposes was obtained from the patients and was approved by the, Ethik-Kommission für klinische Versuche am Menschen und epidemiologische Forschung mit personenbezogenen Daten der Medizinischen Fakultät der Rheinischen Friedrich-Wilhelms-Universität Bonn’ (The ethics committee for clinical trials on humans and epidemiological research with patient-related data of the medical faculty of the Rheinischen-Friedrich-Wilhelms-University Bonn).

All animal experiments were conducted according to the German law of animal protection and in agreement with the approval of the local institutional animal care committees (Landesamt für Natur, Umwelt und Verbraucherschutz, North Rhine-Westphalia (approval ID: AZ-84-02.04.2013-A430). The experiments were conducted in accordance with the International Guiding Principles for Biomedical Research Involving Animals as announced by the Society for the Study of Reproduction.

### Cell culture

GCC cell lines utilized in this study were cultivated as described previously [7]. Briefly, TCam-2 and NCCIT cells were grown in RPMI. The cell lines 2102EP, NT2/D1, 833KE, H12, GCT27, JEG-3 and JAR were grown in DMEM. Both media were supplemented with 10% fetal calf serum (FCS) (PAA, Pasching, Austria), 1% Penicillin /streptomycin (P /S) (PAN, Aidenbach, Germany), 200 mM L-Glutamine (PAN, Aidenbach, Germany). MPAF and ARZ were grown in DMEM (10% FCS, 1% P /S, 200 mM L-Glutamine, 1x non-essential amino acids (PAA, Pasching, Austria), 100 nM ß-Mercaptoethanol (Sigma-Aldrich, Taufkirchen, Germany). TCam-2 [82] cells were kindly provided by Dr. Janet Shipley (Institute of Cancer Research, Sutton, United Kingdom). 2102EP [83], NT2/D1 [84] and NCCIT [85] cells were provided by Prof. Dr. Leendert Looijenga (Erasmus MC, Daniel den Hoed Cancer Center, Josephine Nefkens Institute, Rotterdam, Netherlands). 833KE [86] cells were provided by PD Dr. Beate Köberle (KIT, Karlsruhe, Germany). H12.1 [87] and GCT27 [88] were kindly provided by Dr. Peter Andrews (University of Sheffield, United Kingdom) and obtained from Dr. Thomas Müller (Department of Internal Medicine IV, Oncology and Hematology, Martin-Luther-University of Halle Wittenberg, Halle, Germany). JAR (HTB-144) and JEG-3 (HTB-36) cells were purchased from ATCC. MPAF and ARZ were provided by Dr. Michael Peitz (Life & Brain Center, University of Bonn, Germany).

### Tissue microarrays

Tissue microarrays were assembled and prepared in house after approval by the internal review board. Further information is given in [7].

### Treatment of TCam-2 cells with NOGGIN, LDN193189 and NODAL

TCam-2 cells were seeded 24h before treatment (1 x 10^5^ cells /9,5 cm^2^). 500 ng /ml NOGGIN (diluted in 10 mM HAc) (Abcam, Cambridge, UK), 500 ng /ml LDN193189 (diluted in H_2_O) (Sigma-Aldrich, Taufkirchen, Germany) and 500 ng /ml recombinant NODAL (diluted in 4 mM HCl, 0.1% BSA) (R&D Systems, Wiesbaden, Germany) were added in 2 ml fresh culture medium every second day.

### DNA, RNA and protein isolation

DNA, RNA and proteins were isolated as described previously [47] [89]. DNA was isolated by phenol /chloroform /isoamylalcohol, RNA by TRIzol and proteins by RIPA buffer. DNA and RNA concentrations as well as 260 /280 nm, 260 /230 nm purity ratios were determined by NanoDrop measurement (Peqlab, Erlangen, Germany).

### Western blot

Western blots analyses were performed as described previously [47] [7]. Briefly, the Mini-PROTEAN Electrophoresis Cell and Trans-Blot Turbo system were used (BioRad, Munich, Germany). Gels were blotted onto PVDF membranes. Chemiluminescent signals were detected using ChemiDoc MP Imaging System (BioRad) and band intensities were calculated by Image Lab software (BioRad). Beta-ACTIN was used as housekeeper and for normalization. See S1 Table for antibody details.

### Quantitative RT-PCR

Quantitative RT-PCR (qRT-PCR) was performed as described previously [7]. For first strand synthesis, the RevertAid First Strand cDNA Synthesis Kit manual (Fermentas, St. Leon-Rot, Germany) was used. For PCR, the Maxima SYBR Green qPCR Master Mix (Fermentas, St. Leon-Rot, Germany) was used. PCR was performed using the ViiA 7 Real Time PCR System (Applied Biosystems, distributed by Life Technologies, Carlsbad, CA, USA). At the end of each PCR run, a melting point analysis was performed. *GAPDH* was used as housekeeping gene and for data normalization. Variation of *GAPDH* expression between different experimental setups is very low (S1L Data). See S2 Table for primer sequences.

### Immunohistochemistry and immunofluorescence staining

Immunohistochemistry (IHC) was performed as published previously [47] [7]. Tumor tissues were dissected, fixed in 4% formalin overnight and processed in paraffin wax. Signal detection was performed semiautomatically in the Autostainer 480 S (Medac, Hamburg, Germany). Nuclei were stained by hematoxylin. Immunofluorescence staining (IF) was performed as published [15] [47]. Nuclei were counterstained by Hoechst 33342. See S1 Table for antibody details and dilution ratios.

### Sodium bisulfite sequencing

Sodium bisulfite sequencing was performed as described previously [89]. Briefly, 500 ng of DNA were sodium bisulfite converted using the ‘EZ DNA-Methylation Gold kit’ (Zymo Research, Freiburg, Germany). See S2 Table for primer details.

### Xenotransplantation of GCC cell lines

Xenotransplantation was performed as described previously [48]. 1 x 10^7^ cells in 500 μl of 4°C cold Matrigel (BD, Heidelberg, Germany) were injected into the flank of CD1 nude mice.

### Illumina HT-12v4 expression array and Infinium 450K methylation array

RNA quality was checked for degradation via gel electrophoresis in a BioAnalyzer 2100 (Agilent Technologies, Waldbronn, Germany) using RNA 6000 nano lab chips. DNA was sodium-bisulfite converted using the EZ DNA Methylation kit (Zymo Research, Freiburg, Germany). Samples were processed on Illuminas' (San Diego, California, USA) human, HT-12v4’ and human, Infinium Methylation 450k Bead Chips’.

A subset quantile normalization approach developed by N. Touleimat & J. Tost was applied [90]. This approach includes signal correction for the adjustment of the color balance and background level correction as well as the Infinium I/Infinium II shift correction between sample normalization. Technical quality parameters such as hybridization, extension, bisulfite conversion and specificity were evaluated using the, Genome Studio’ software. Beta-value signal distributions were inspected by density plots. Data was analyzed using, Bioconductor R’ (www.bioconductor.org). To increase performance in terms of detection and true positive rate of highly methylated and unmethylated CpG-sites, beta-values were transformed to M-values [91]. Differentially methylated loci were identified using a t-test. p-values were corrected for multiple testing using the Benjamini-Hochberg correction. The expression values were quantile normalized using the, limma’-software-package (‘Linear Models for Microarray Data’, www.bioconductor.org). For inverse correlation analysis of methylation and gene expression data, methylation at CpGs and gene expression transcripts were mapped to the same gene identifiers. Inverse correlation was calculated using the Pearson correlation coefficient and p-values for association were corrected for multiple testing using the Benjamini-Hochberg correction. Microarray data sets are publically available via GEO (ncbi.nlm.nih.gov/geo/) (GSE60698, GSE60787).

### Affymetrix cDNA microarray analysis of GCC tissues

The whole procedure has already been published [15]. The array was reanalyzed in context of this study. Normalized gene expression intensities of averaged seminomas were substracted from averaged intensities of EC tissues (Seminoma group) and normalized gene expression intensities of averaged ECs were substracted from averaged intensities of seminoma tissues (EC group).

### BDPC, STRING and GeneTrail analysis, Circos and Venn diagrams

BDPC analysis and STRING protein-protein-interaction prediction were performed online using default settings (services.ibc.uni-stuttgart.de/BDPC) (string-db.org) [92] [93]. GeneTrail-based GO analysis was also performed online using default settings (genetrail.bioinf.uni-sb.de) [94]. Circos diagrams were generated using ‘Circos Table Viewer’ (mkweb.bcgsc.ca/tableviewer) [95] and Venn diagrams were generated using ‘Venny’ (bioinfogp.cnb.csic.es/tools/venny).

## Supporting Information

S1 Fig 5mC distribution across the genome of transiting TCam-2. Averaged 5mC levels of all differentially methylated genes at indicated regulatory genomic regions during SET.
(TIF)

S2 Fig Dynamics of 5mC in CpG-island- and open-sea-context. (A, B) 5mC levels of CpGs in CpG-island- (A) and open sea-context (B) across different genomic regions. TCam-2 in vivo 6w and 2102EP data was normalized to TCam-2 in vitro (blue lines).
(TIF)

S3 Fig 5mC dynamics across chromosomes and BDPC analysis of 5mC data. (A) Numbers of differentially methylated CpGs (0–40%, 41–80%, > 81%) between in vitro cultivated and xenografted TCam-2 compared to 2102EP cells. (B) BDPC analysis of xenografted TCam-2 /2102EP demonstrates that xenografted TCam-2 (4w, 6w) cluster more closely to 2102EP samples during SET regarding 5mC.
(TIF)

S4 Fig Validation of selected deregulations in Gex and 5mC. (A) qRT-PCR analysis of indicated genes during xenografting of TCam-2 (1–6 weeks). (B) IHC of indicated genes in TCam-2 cells xenografted for 1–2 weeks. (C) Sodium bisulfite sequencing of the *GDF3* promotor in parental TCam-2 /2102EP and TCam-2 /2102EP xenografted for 4 weeks. Empty circles represent unmethylated CpGs and filled circles methylated CpGs.
(TIF)

S5 Fig Interactive network prediction and verification of Gex data in EC cell lines. (A, B) STRING-based interaction prediction of genes commonly upregulated in TCam-2 1 (A) and 6 (B) weeks after xenografting. (C) qRT-PCR analysis of indicated seminoma and EC markers in parental TCam-2 and EC cell lines (2101EP, NCCIT, NT2/D1, 833KE, H12).
(TIF)

S6 Fig Signaling pathways that drive reprogramming of TCam-2 cells into an EC. (A) cDNA microarray expression data of indicated signaling pathway-associated genes during SET and in 2102EP control cells. (B) Western blot analysis of SOX2 and pSMAD1 /5 expression in in vitro cultivated and xenografted TCam-2 cells (4 weeks). (C, D) Western blot analysis of SMAD1 /5-phosphorylation in TCam-2 treated with NOGGIN (C) and LDN193189 (D) or corresponding solvents. (E) qRT-PCR analysis of indicated genes in TCam-2 cells treated with the BMP inhibitor LDN193189 for 48–96 h. (F) Western blot analysis of SMAD2 /3-phosphorylation 72 h after treatment of TCam-2 cells with recombinant NODAL or the solvent. (G) qRT-PCR analysis of indicated genes in TCam-2 cells treated for 24 and 72 h with recombinant NODAL.
(TIF)

S7 Fig GCC-TMA-IHCs and western blot analysis of ZIC3 expression. (A) Examples of ID1, TFAP2C, SOX2 and beta-CATENIN IHC in CIS, seminoma and EC tissues. Scale bars: 100 μm. (B) Western blot analysis of ZIC3 and SOX2 expression in indicated GCC cell lines and human fibroblasts. (C) XY-diagrams illustrating the correlation /reciprocal correlation of *ZIC3* to *SOX2*, *SOX17* and *NODAL* in GCC tissues.
(TIF)

S1 Data Summarized cDNA expression and DNA methylation results. (XLSX)

S1 Table Antibodies used in this study. (XLSX)

S2 Table Oligonucleotides used in this study. (XLSX)
