# Supplementary figures and images for: BMP Inhibition in Seminomas Initiates Acquisition of Pluripotency via NODAL Signaling Resulting in Reprogramming to an Embryonal Carcinoma
Source: PLoS Genet. 2015 Jul 30;11(7):e1005415. doi: 10.1371/journal.pgen.1005415 (PMC4520454; doi:10.1371/journal.pgen.1005415)

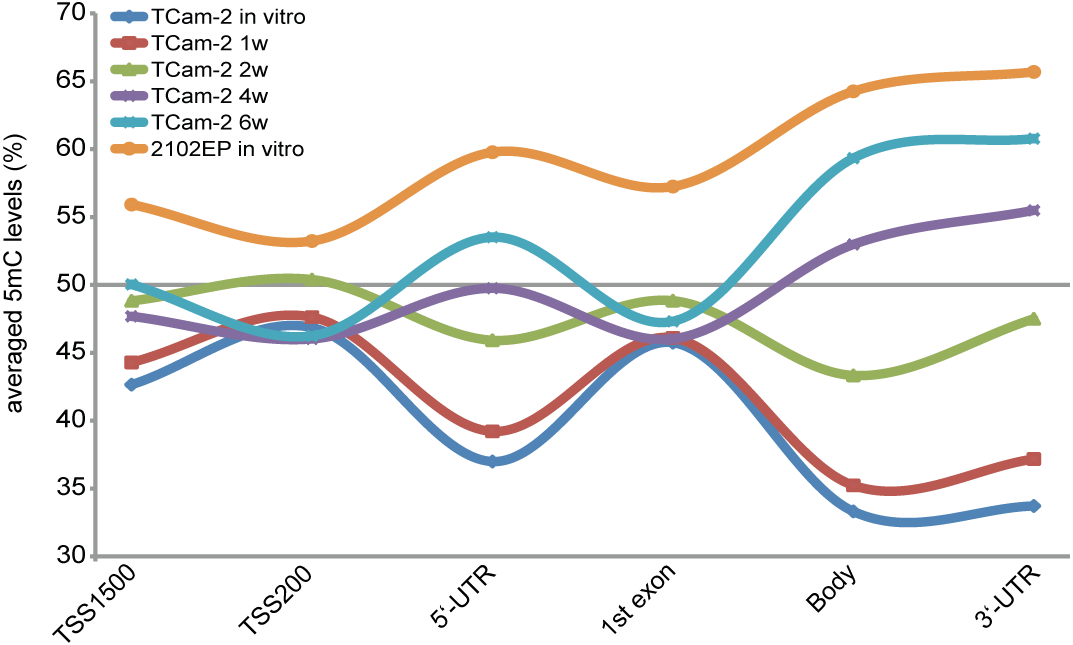

Supplement: S1 Fig — Averaged 5mC levels of all differentially methylated genes at indicated regulatory genomic regions during SET. (TIF) [file pgen.1005415.s001.tif]

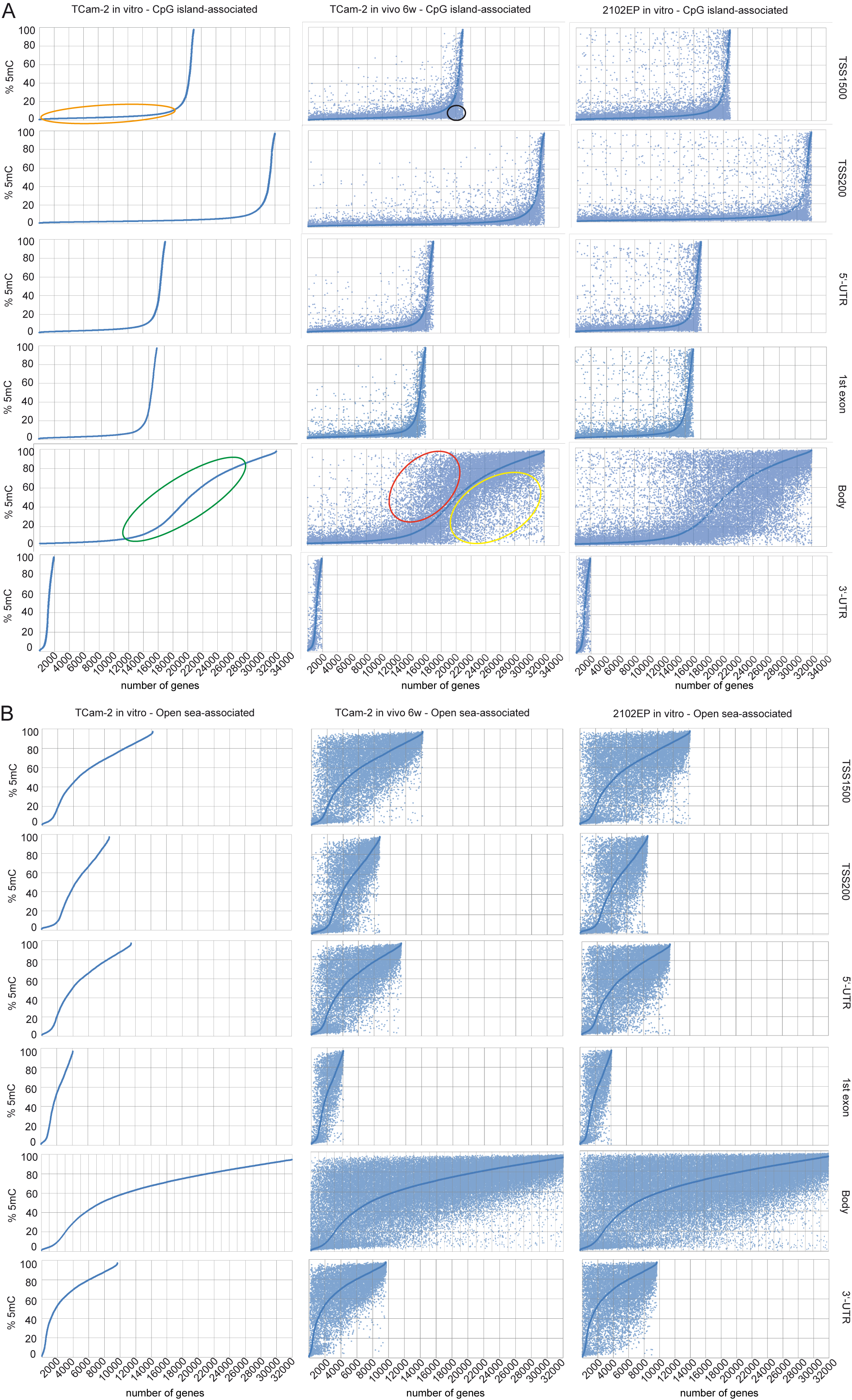

Supplement: S2 Fig — (A, B) 5mC levels of CpGs in CpG-island- (A) and open sea-context (B) across different genomic regions. TCam-2 in vivo 6w and 2102EP data was normalized to TCam-2 in vitro (blue lines). (TIF) [file pgen.1005415.s002.tif]

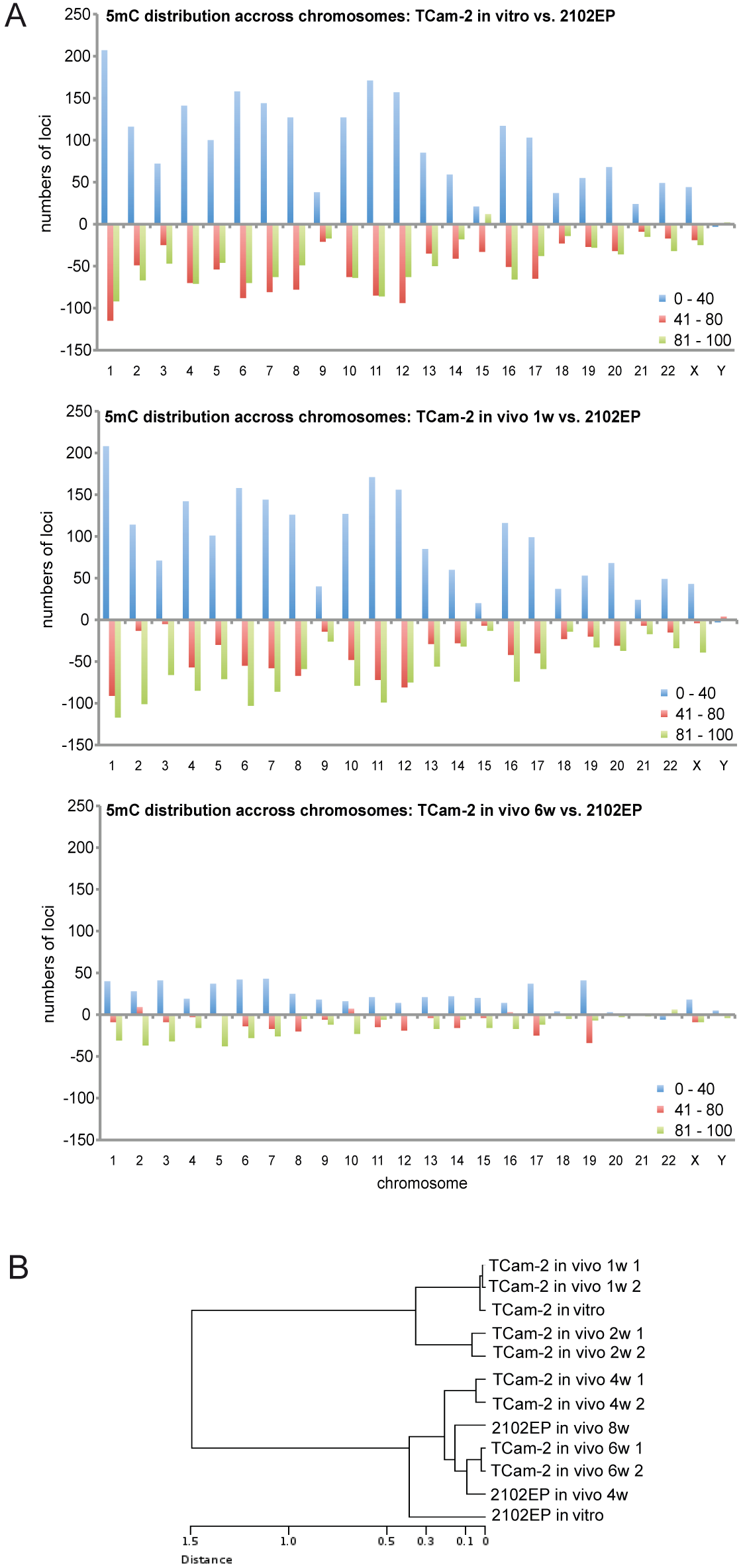

Supplement: S3 Fig — (A) Numbers of differentially methylated CpGs (0–40%, 41–80%, > 81%) between in vitro cultivated and xenografted TCam-2 compared to 2102EP cells. (B) BDPC analysis of xenografted TCam-2 /2102EP demonstrates that xenografted TCam-2 (4w, 6w) cluster more closely to 2102EP samples during SET regarding 5mC. (TIF) [file pgen.1005415.s003.tif]

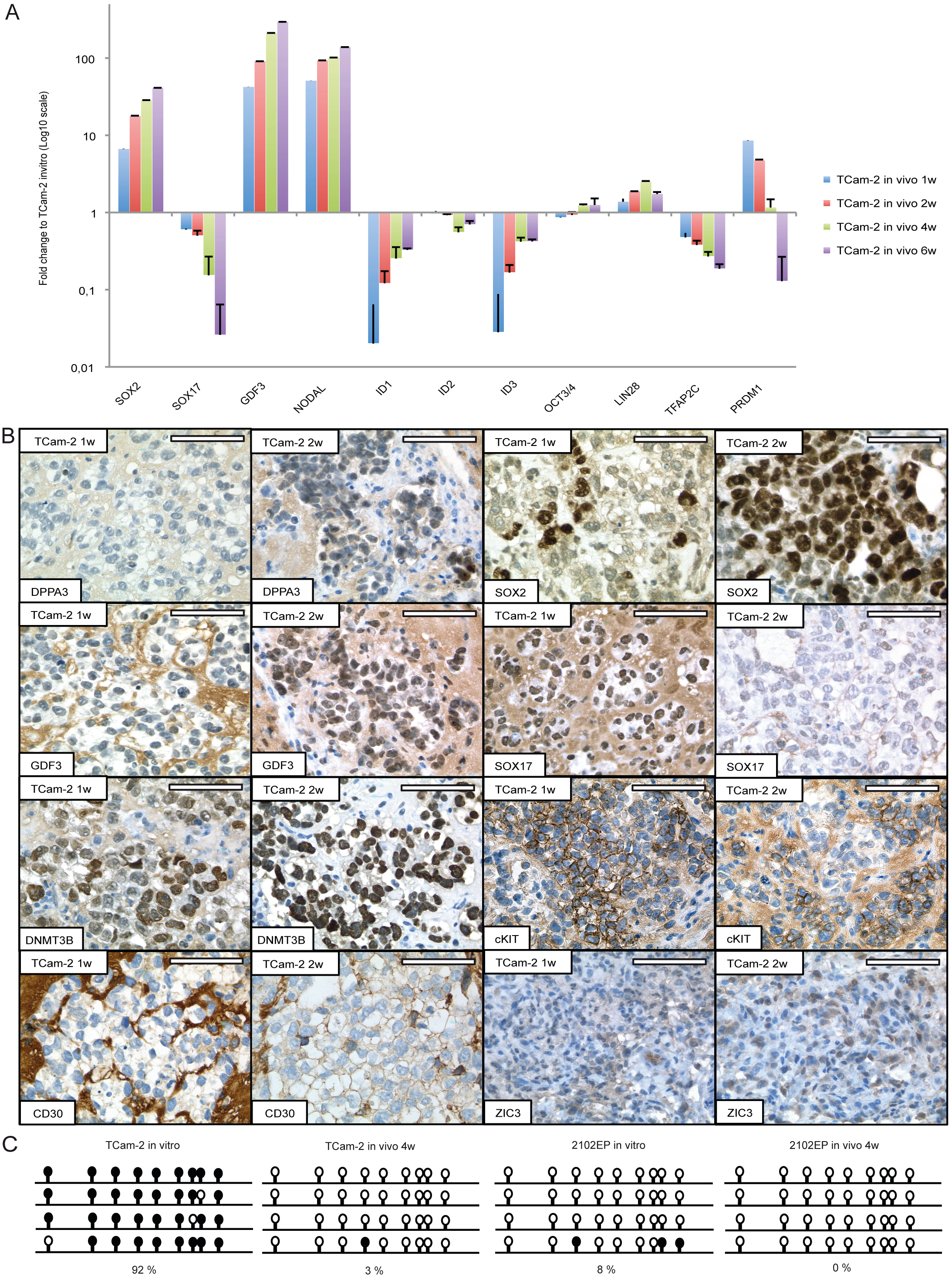

Supplement: S4 Fig — (A) qRT-PCR analysis of indicated genes during xenografting of TCam-2 (1–6 weeks). (B) IHC of indicated genes in TCam-2 cells xenografted for 1–2 weeks. (C) Sodium bisulfite sequencing of the GDF3 promotor in parental TCam-2 /2102EP and TCam-2 /2102EP xenografted for 4 weeks. Empty circles represent unmethylated CpGs and filled circles methylated CpGs. (TIF) [file pgen.1005415.s004.tif]

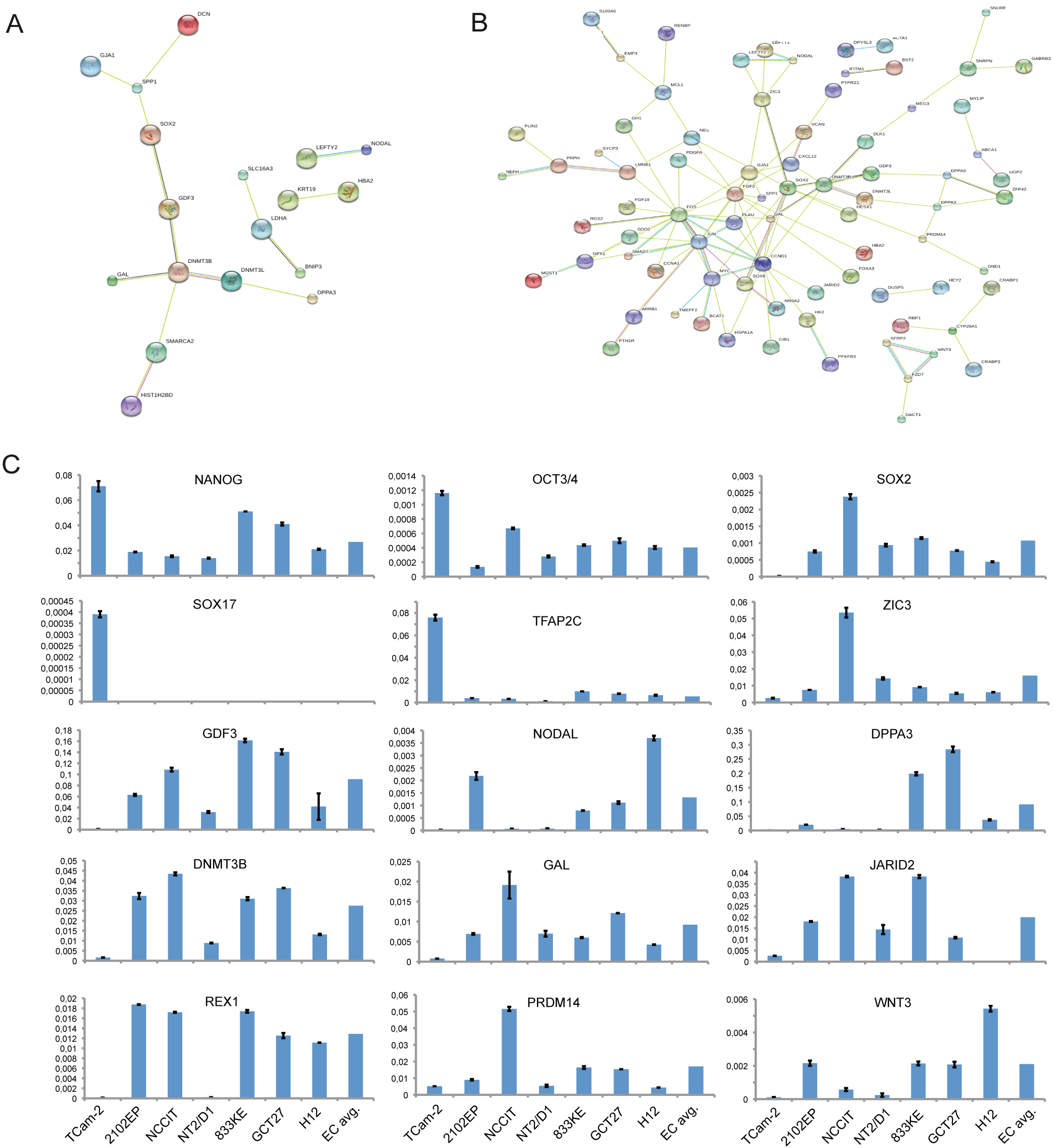

Supplement: S5 Fig — (A, B) STRING-based interaction prediction of genes commonly upregulated in TCam-2 1 (A) and 6 (B) weeks after xenografting. (C) qRT-PCR analysis of indicated seminoma and EC markers in parental TCam-2 and EC cell lines (2101EP, NCCIT, NT2/D1, 833KE, H12). (TIF) [file pgen.1005415.s005.tif]

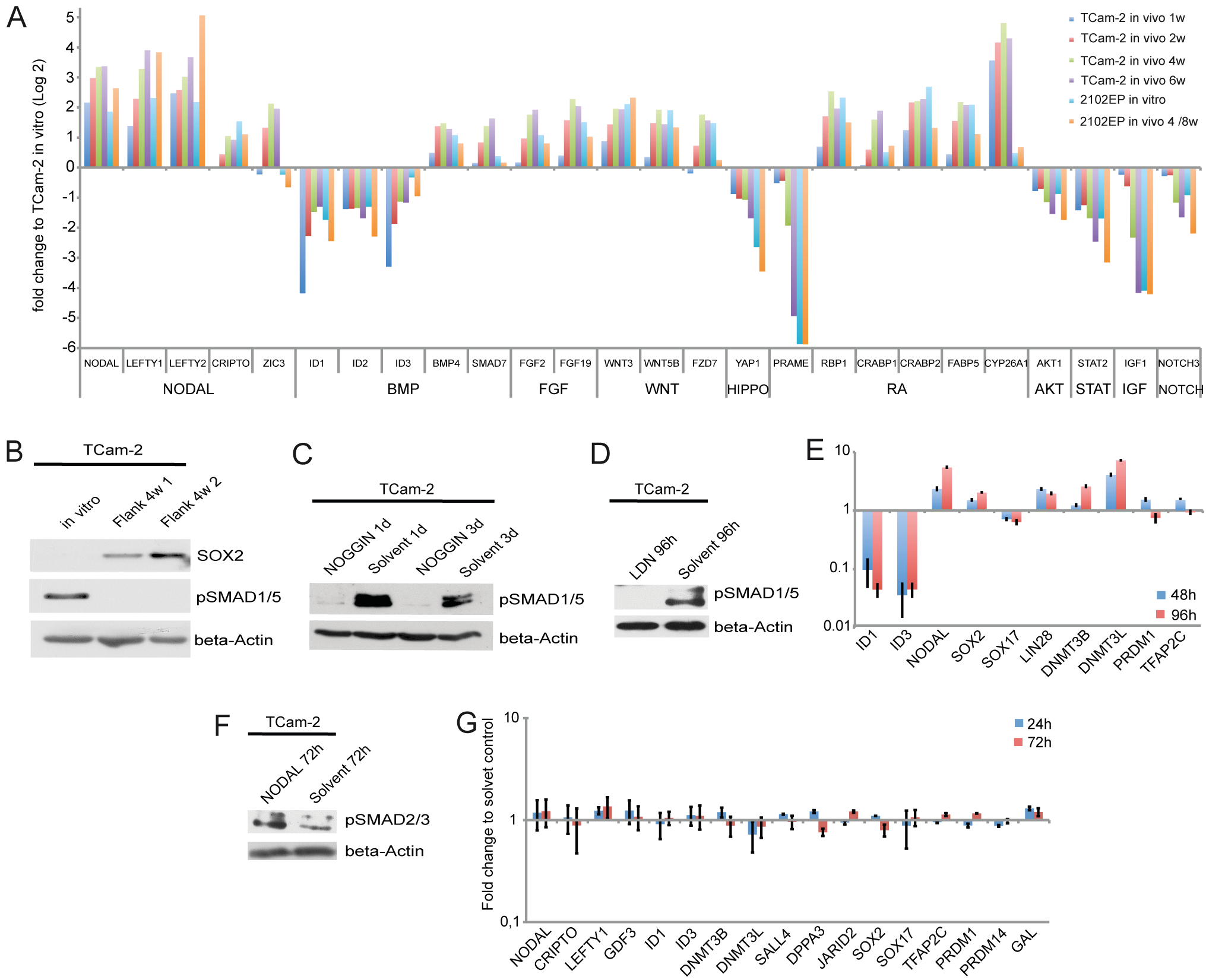

Supplement: S6 Fig — (A) cDNA microarray expression data of indicated signaling pathway-associated genes during SET and in 2102EP control cells. (B) Western blot analysis of SOX2 and pSMAD1 /5 expression in in vitro cultivated and xenografted TCam-2 cells (4 weeks). (C, D) Western blot analysis of SMAD1 /5-phosphorylation in TCam-2 treated with NOGGIN (C) and LDN193189 (D) or corresponding solvents. (E) qRT-PCR analysis of indicated genes in TCam-2 cells treated with the BMP inhibitor LDN193189 for 48–96 h. (F) Western blot analysis of SMAD2 /3-phosphorylation 72 h after treatment of TCam-2 cells with recombinant NODAL or the solvent. (G) qRT-PCR analysis of indicated genes in TCam-2 cells treated for 24 and 72 h with recombinant NODAL. (TIF) [file pgen.1005415.s006.tif]

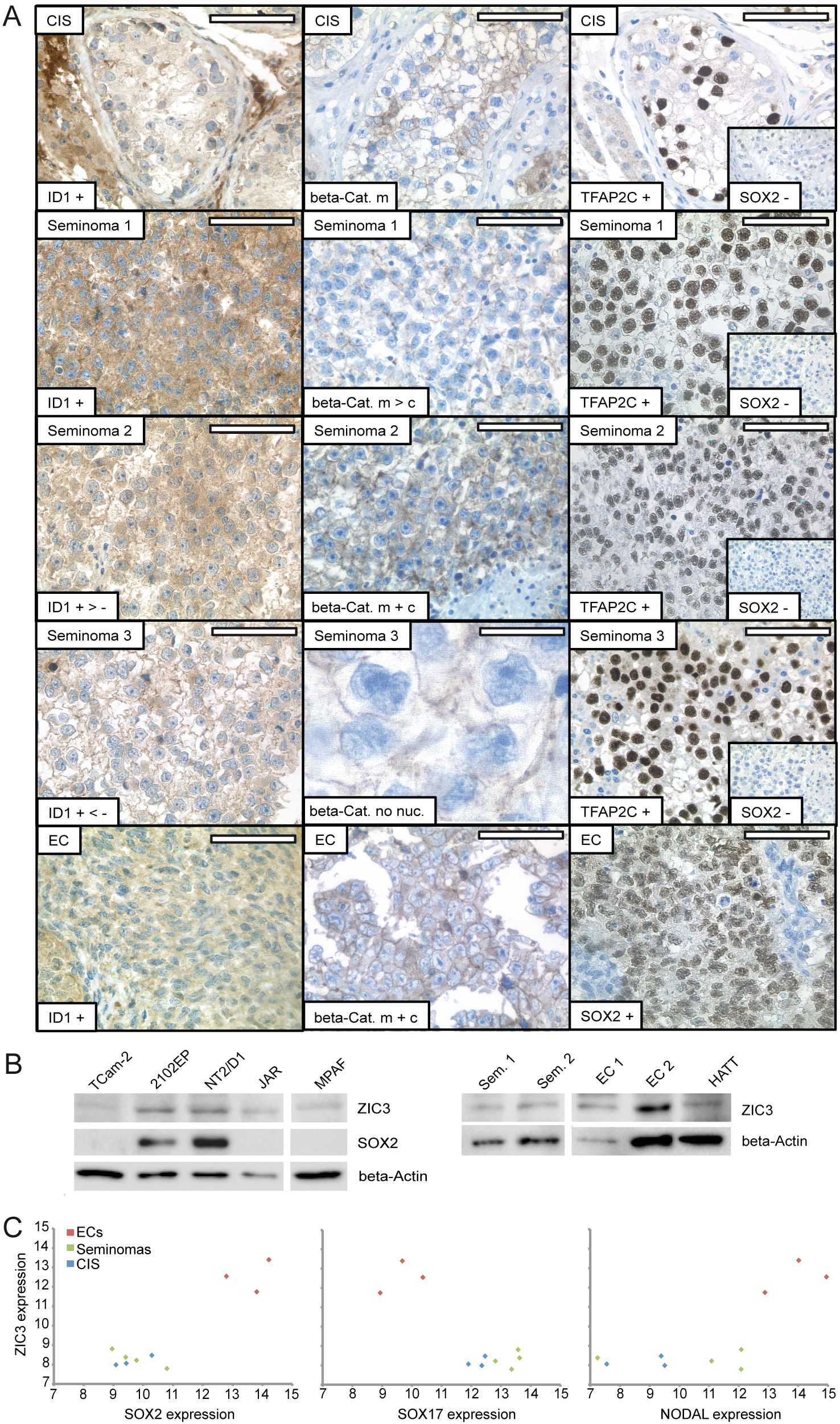

Supplement: S7 Fig — (A) Examples of ID1, TFAP2C, SOX2 and beta-CATENIN IHC in CIS, seminoma and EC tissues. Scale bars: 100 μm. (B) Western blot analysis of ZIC3 and SOX2 expression in indicated GCC cell lines and human fibroblasts. (C) XY-diagrams illustrating the correlation /reciprocal correlation of ZIC3 to SOX2, SOX17 and NODAL in GCC tissues. (TIF) [file pgen.1005415.s007.tif]
